# Tropomyosin1 isoforms underlie epithelial to mesenchymal plasticity, metastatic dissemination, and resistance to chemotherapy in high-grade serous ovarian cancer

**DOI:** 10.1038/s41418-024-01267-9

**Published:** 2024-02-16

**Authors:** Tong Xu, Mathijs P. Verhagen, Miriam Teeuwssen, Wenjie Sun, Rosalie Joosten, Andrea Sacchetti, Patricia C. Ewing-Graham, Maurice P. H. M. Jansen, Ingrid A. Boere, Nicole S. Bryce, Jun Zeng, Herbert R. Treutlein, Jeff Hook, Edna C. Hardeman, Peter W. Gunning, Riccardo Fodde

**Affiliations:** 1 Department of Pathology, Erasmus University Medical Center, Rotterdam, The Netherlands; 2 Institut Curie, Laboratory of Genetics and Developmental Biology, Paris, France; 3 Department of Medical Oncology, Erasmus University Medical Center, Rotterdam, The Netherlands; 4 School of Biomedical Sciences, Faculty of Medicine and Health, The University of New South Wales, Sydney, New South Wales Australia; 5 Computist Bio-NanoTech, Scoresby, VIC 3179 Australia; 6 Present Address: Elisabeth-TweeSteden Ziekenhuis (ETZ), Tilburg, The Netherlands; 7 Present Address: The Victor Chang Cardiac Research Institute, Darlinghurst, NSW Australia; 8 Present Address: Sanoosa Pty. Ltd, Moonee Ponds, VIC 3039 Australia

**Keywords:** Metastasis, Cancer microenvironment

## Abstract

Phenotypic plasticity, defined as the ability of individual cells with stable genotypes to exert different phenotypes upon exposure to specific environmental cues, represent the quintessential hallmark of the cancer cell en route from the primary lesion to distant organ sites where metastatic colonization will occur. Phenotypic plasticity is driven by a broad spectrum of epigenetic mechanisms that allow for the reversibility of epithelial-to-mesenchymal and mesenchymal-to-epithelial transitions (EMT/MET). By taking advantage of the co-existence of epithelial and quasi-mesenchymal cells within immortalized cancer cell lines, we have analyzed the role of EMT-related gene isoforms in the regulation of epithelial mesenchymal plasticity (EMP) in high grade serous ovarian cancer. When compared with colon cancer, a distinct spectrum of downstream targets characterizes quasi-mesenchymal ovarian cancer cells, likely to reflect the different modalities of metastasis formation between these two types of malignancy, i.e. hematogenous in colon and transcoelomic in ovarian cancer. Moreover, upstream RNA-binding proteins differentially expressed between epithelial and quasi-mesenchymal subpopulations of ovarian cancer cells were identified that underlie differential regulation of EMT-related isoforms. In particular, the up- and down-regulation of RBM24 and ESRP1, respectively, represent a main regulator of EMT in ovarian cancer cells. To validate the functional and clinical relevance of our approach, we selected and functionally analyzed the Tropomyosin 1 gene (*TPM1*), encoding for a protein that specifies the functional characteristics of individual actin filaments in contractile cells, among the ovarian-specific downstream AS targets. The low-molecular weight *Tpm1.8/9* isoforms are specifically expressed in patient-derived ascites and promote invasion through activation of EMT and Wnt signaling, together with a broad spectrum of inflammation-related pathways. Moreover, *Tpm1.8/9* expression confers resistance to taxane- and platinum-based chemotherapy. Small molecule inhibitors that target the *Tpm1* isoforms support targeting Tpm1.8/9 as therapeutic targets for the development of future tailor-made clinical interventions.

## Introduction

Epithelial ovarian cancer (EOC) is the leading cause of death amongst gynecologic malignancies due to its high case-to-fatality ratio [1, 2]. EOC generally becomes manifest at advanced disease stages, i.e. when metastases have already spread to pelvic organs (stage II), the abdomen (stage III), or beyond the peritoneal cavity (stage IV) [3]. Based on the underlying genetic defects, two main EOC subtypes have been recognized [4]. Type I tumors are slow growing, mostly restricted to the ovary, and thought to arise from well-differentiated precursor lesions called “borderline” tumors. They are further subdivided into low-grade serous, mucinous, clear cell, and endometrioid subtypes. Mutations in *KRAS*, *BRAF*, *PTEN*, and *CTNNB1* (β-catenin) earmarks type I EOCs, often together with a relatively stable karyotype. High-grade serous (HGSOC) and undifferentiated carcinomas are type II EOCs and are frequently characterized by *TP53* mutations and by aneuploidy [4]. HGSOC represents the most malignant and common ovarian cancer type accounting for up to 70% of all cases with poor prognosis and survival [5].

Of note, EOC is the only cancer type where no physical barrier exists between primary lesion and the main metastatic site, i.e. the intraperitoneal cavity. The dissemination of ovarian cancer cells results in their adhesion to intra-abdominal organs and the peritoneum [6] eventually leading to ascites accumulation due to the obstruction of lymphatic vessels [7].

Epithelial-to-mesenchymal (EMT) and mesenchymal-to-epithelial transitions (MET) are thought to underlie local dissemination and distant metastasis in the majority of epithelial malignancies, including ovarian cancer where, as mentioned above, the absence of a physical barrier would suggest alternative shedding mechanisms [8]. EMT/MET are regulated by a broad spectrum of epigenetic mechanisms involving chromatin remodeling due to histone methylation/acetylation, non-coding RNAs, promoter DNA methylation, and post-transcriptional mechanisms such as alternative splicing (AS) [9]. AS occurs in the majority of human genes and represents a major determinant of protein diversity [10]. A broad spectrum of RNA-binding proteins (RBPs) regulate splicing by recognizing specific sequences within (pre)mRNA and are known to play a role in AS through their differential expression among different tissues in homeostasis and disease [11–14]. RBP-driven alternative splicing is thought to play a crucial role in cancer metastasis, as well as in EMT/MET [15]. Among the various RBPs, ESRP1 (epithelial splicing regulatory protein 1) was shown to regulate splicing of multiple downstream targets during EMT in different cancer types [16].

Previously, we have shown that *ESRP1*, together with other RBP-coding genes, are differentially expressed between epithelial and quasi-mesenchymal colon cancer cells as the result of their role in the regulation of alternative splicing downstream of EMT [17]. Among several AS targets, the *CD44* and *NUMB* genes were shown to play relevant functional roles to promote EMT and phenotypic plasticity in colon cancer. Here, we have employed a similar approach to study the role of alternative splicing and alternative promoter usage in EOC metastasis. Systematic analysis of the downstream EMT isoform targets only revealed a small overlap with what was previously identified in colon cancer [17]. Accordingly, the majority of the targets were ovarian cancer-specific as illustrated by the Tropomyosin 1 (*TPM1*) gene that encodes for various actin-binding protein isoforms that specify the functional characteristics of individual actin filaments in muscle and non-muscle cells [18]. *TPM1* was previously shown to suppress tumor development in multiple cancer types through distinct mechanisms [19, 20]. Here, we established that the low molecular weight isoforms *Tpm1.8/9* play a key role in EMT, Wnt signaling, and chemo-resistance in ovarian cancer and represent a candidate target for future therapies as shown by the in vitro results obtained both by isoform-specific siRNA and small molecule inhibitors.

## Results

### A subpopulation of quasi-mesenchymal cells co-exists with epithelial cells in high-grade serous ovarian cancer

**Fig. 1:**
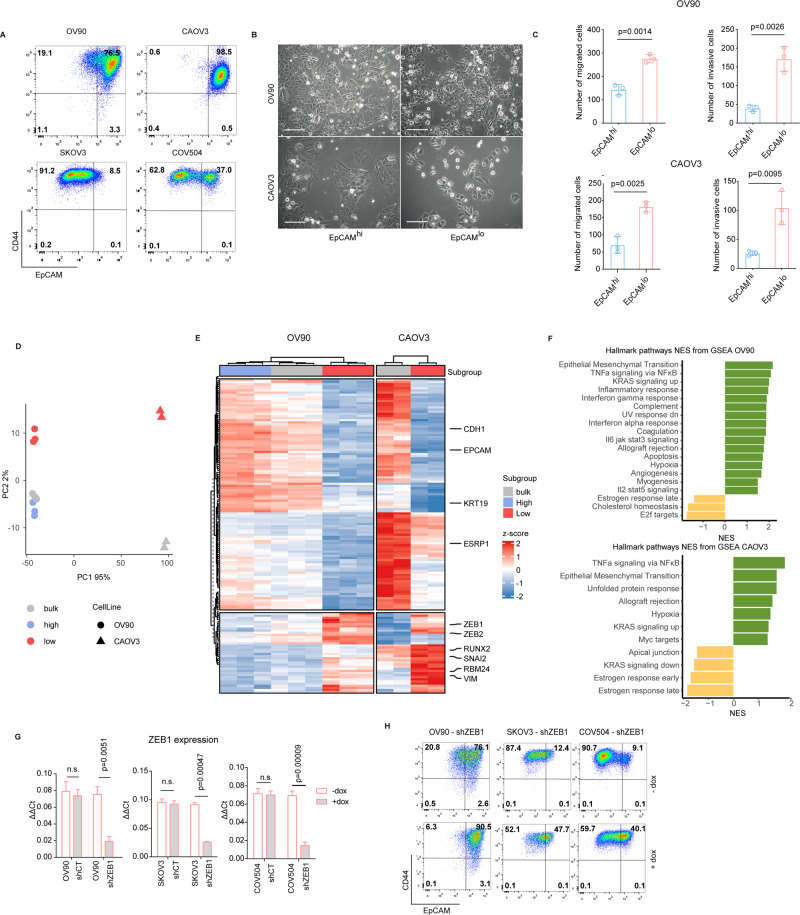
A subpopulation of quasi-mesenchymal cells co-exists with epithelial cells in HGS ovarian cancer cell lines. **A** FACS analysis of the ovarian cancer cell lines OV90, CAOV3, SKOV3, and COV504 with antibodies directed against CD44 and EpCAM. EpCAM/CD44 positive and negative areas were defined as previously described [17, 21] using multiple isotype controls and are shown by the quadrants in the plots. Using specific gates, cells were separated in CD44^hi^EpCAM^hi^ and CD44^hi^EpCAM^lo^ subpopulations. The percentages of CD44^hi^EpCAM^lo^ and CD44^hi^EpCAM^hi^ cells within each cell line are depicted in each quadrant. Notably, as previously observed for SW480 and HCT116, the ovarian cancer cell lines revealed a continuum of different EpCAM and CD44 expression levels with a large EpCAM^hi^ (or EpCAM^lo^ as in the case of SKOV3) cluster followed by a tail of gradually decreasing (increasing for SKOV3) EpCAM levels. By applying the indicated gates, cells were sorted into EpCAM^hi^ and EpCAM^lo^ subpopulations. Graphs show representative analysis from an individual experiment. **B** Phase contrast microscopy images of sorted EpCAM^hi^ and EpCAM^lo^ cells from EpCAM^hi^ and EpCAM^lo^ OV90 and CAOV3 sorted cells. Scale bar: 100 μm. While EpCAM^hi^ cells show characteristic epithelial morphology, EpCAM^lo^ cells showed a more spindle- and mesenchymal-like appearance. Scale bar: 100 µm. **C** Transwell migration assay (upper graph) and invasion assay (lower graph) of EpCAM^hi^ (blue bar) and EpCAM^lo^ (red bar) OV90 and CAOV3 cells. Each bar symbolizes the mean ± SD. **D** Principal component analysis (PCA) of the RNAseq profiles of EpCAM^hi^ and EpCAM^lo^ cells from the OV90 and CAOV3 lines. **E** Heatmap of common differentially expressed gene among EpCAM^hi/lo^ and bulk subpopulations from the OV90 and CAOV3 cell lines (abs LFC > 1.5, *P* value < 0.01). Complete-linkage hierarchical clustering with split by k-means (k = 2) clustering was used. **F** Hallmark pathways based on the Gene Set Enrichment Analysis (GSEA) of OV90 and CAOV3 EpCAM^lo^ expression profiles compared with EpCAM^hi^. Plots show only significantly expressed pathways, with a normalized enrichment score (NES) > 1 and *P* value < 0.05. **G** RT-qPCR expression analysis of *ZEB1* in OV90, SKOV3 and COV504 transduced with an inducible control (shCT) and with a *ZEB1*-shRNA (shZEB1) construct. shRNA expression was induced with 1 μg/mL of doxycycline. GAPDH expression was used as control. Each bar represents the mean ± SD. *P* value is indicated. **H** FACS analysis of the OV90, SKOV3 and COV504 cell lines transfected with the sh*ZEB1* and control constructs using antibodies against CD44 and EpCAM. Cells were induced with 1 μg/mL doxycycline for 72 h prior to the FACS analysis. The percentages of EpCAM^lo^ and EpCAM^hi^ cells within each cell line are depicted in each quadrant.

Following the experimental strategy previously adopted to identify subpopulations of quasi-mesenchymal cells in immortalized colon cancer cell lines [17, 21], we analyzed the HGSOC cell lines OV90, SKOV3, COV504, and CAOV3 by FACS with CD44 and EpCAM antibodies. As shown in Fig. 1A, distinct distributions of CD44^hi^EpCAM^hi^ and CD44^hi^EpCAM^lo^ cells (from here on referred to as EpCAM^hi^ and EpCAM^lo^, respectively) earmarked each line albeit in different percentages. Upon sorting and short-term culturing, EpCAM^lo^ cells showed a mesenchymal-like morphology, in contrast with the epithelial appearances of their EpCAM^hi^ counterparts (Fig. 1B). Accordingly, EpCAM^lo^ cells showed increased migration and invasion capacity in trans-well assays (Fig. 1C).

In order to elucidate the global gene expression profiles of the two distinct subpopulations of ovarian cancer cells, RNAseq analysis was carried out on the EpCAM^hi/lo^ cells sorted by FACS from the OV90 and CAOV3 lines. Principal component analysis (PCA) by multidimensional scaling (MDS) revealed a clear separation of the quasi-mesenchymal EpCAM^lo^ cells from their epithelial counterpart in the second dimension (Fig. 1D). Unsupervised clustering of the RNAseq data highlighted differentially expressed genes between the EpCAM^lo^ and EpCAM^hi^ subpopulations from the two cell lines (Fig. 1E). Among these genes, different EMT transcription factors (EMT-TFs; *ZEB1/2*, *RUNX2*, *SNAI2*), EMT target genes (*CDH1*, *VIM*), and EMT-related RBPs (*ESRP1*, *RBM24*) earmarked EpCAM^lo^ cells in both OV90 and CAOV3. Accordingly, pathway analysis (PA) of the genes whose expression earmarks the quasi-mesenchymal ovarian cancer cells revealed significant associations with EMT, KRAS signaling, and several TNF- and interferon-related inflammatory pathways (Fig. 1F). Pathways with a normalized enrichment score (NES) > 1 and *P* value < 0.05 were labeled as significant. To confirm the central role played by EMT in the establishment of the EpCAM^lo^ identity as indicated by the PA analysis, *ZEB1* expression was downregulated by shRNA in the OV90, SKOV3, and COV504 cell lines (Fig. 1G). As expected, FACS analysis of these cells shows that knockdown of the differentially expressed EMT-TF results in a substantial reduction of the EpCAM^lo^ subpopulation (Fig. 1H).

**Fig. 2:**
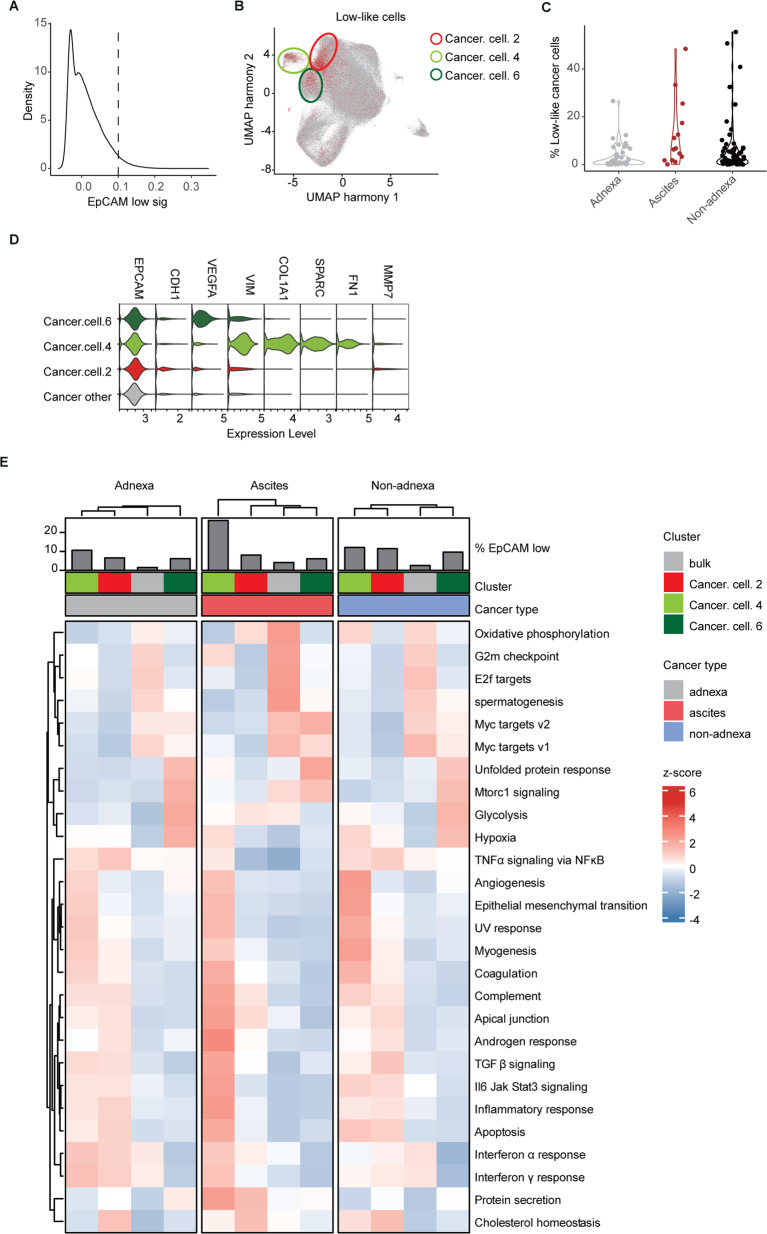
EpCAM^lo^ cells co-exist with epithelial cells in high-grade serous ovarian cancer. **A** Density plot shows the distribution of patient-derived ovarian cancer cells (scRNAseq data from Vazquez-Garcia et al. [22]) earmarked by expression of the EpCAM^lo^ signature. The threshold was set as ≥0.1 for the subsequent analyses. The EpCAM^lo^ signature was defined by genes that were identified as upregulated EpCAM^lo^ cells in both CAOV3 and OV90. EpCAM^lo^ upregulated genes were selected after differential expression analysis with EpCAM^hi^/bulk cells using the cut-offs log2FC > 1.5 and padj <0.05. **B** UMAP plot of patient-derived ovarian cancer cells. Cells positive for the EpCAM^lo^ signature are highlighted in red and show enrichment within three clusters (#2, #4, and #6) labeled as EMT-like in the Vazquez-Garcia et al. study [22]. Please note that a substantial fraction of the EpCAM^lo^-like cells appears to fall outside these clusters and is distributed throughout the UMAP. **C** Violin plots showing the distribution of EpCAM^lo^-like cells (according to z-score) in different anatomical localization of ovarian cancers (adnexa, ascites, and non-adnexa). **D** Violin plots showing the expression levels of EMT-like signature of single cell cluster 2, 4 and 6. **E** Heatmap of hallmark gene sets across the EpCAMlo-like clusters in primary ovarian cancers (adnexa), ascites, and metastases (non-adnexa) based on the GSEA of three EpCAM^lo^-like clusters and bulk (*p* val <0.05). Complete-linkage hierarchical clustering was used.

To demonstrate the clinical relevance of the above data obtained from immortalized cancer cell lines, publicly available single-cell RNAseq data from 42 patient-derived ovarian cancers [22] were employed to search for quasi-mesenchymal cells reminiscent of the EpCAM^lo^ subpopulation. To this aim, we took advantage of the EpCAM^lo^ signature derived from the above RNAseq analysis of the OV90 and CAOV3 cell lines (Supplementary Table 1). After classifying patient-derived cancer cells according to the EpCAM^lo^ signature (Fig. 2A), approx. 60% were found to cluster into one of the three subpopulations of HGS ovarian cancer cells defined in the original study by Vazquez-Garcia et al. [22] as EMT-like (labeled as #2, #4, and #6) (Fig. 2B). Of note, a substantial fraction of the EpCAM^lo^-like cells fell outside the three clusters indicative of large heterogeneity of cellular identities reminiscent of the quasi-mesenchymal state in the cell lines. As shown in Fig. 2C, cells from within the EpCAM^lo^-like clusters were more represented in metastases and ascites (i.e. non-adnexa including omentum, peritoneum, etc.) when compared with primary tumors. We then evaluated module scores from the Hallmark gene sets across the EpCAM^lo^-like clusters in primary ovarian cancers (adnexa), ascites, and metastases (non-adnexa). As shown in Fig. 2D, E, EMT-like signature and inflammatory signaling pathways (TNFα, IL6/Jak/Stat3, IFN, and TGFβ) were significantly upregulated in EpCAM^lo^-like clusters, in particular in cluster #4 in ascites and metastatic lesions.

Collectively, these results indicate that, HGSOC cell lines encompass subpopulations of quasi-mesenchymal cells endowed with increased motility and invasive capacity, and characterized by EMT-TFs the expression of which is central to their cellular identity. Similar subpopulations of ovarian cancer cells are found in patient-derived primary and metastatic lesions, and in malignant ascites.

### Differential expression of RNA-binding proteins underlies alternative splicing of a subset of target genes in quasi-mesenchymal ovarian cancer cells

**Fig. 3:**
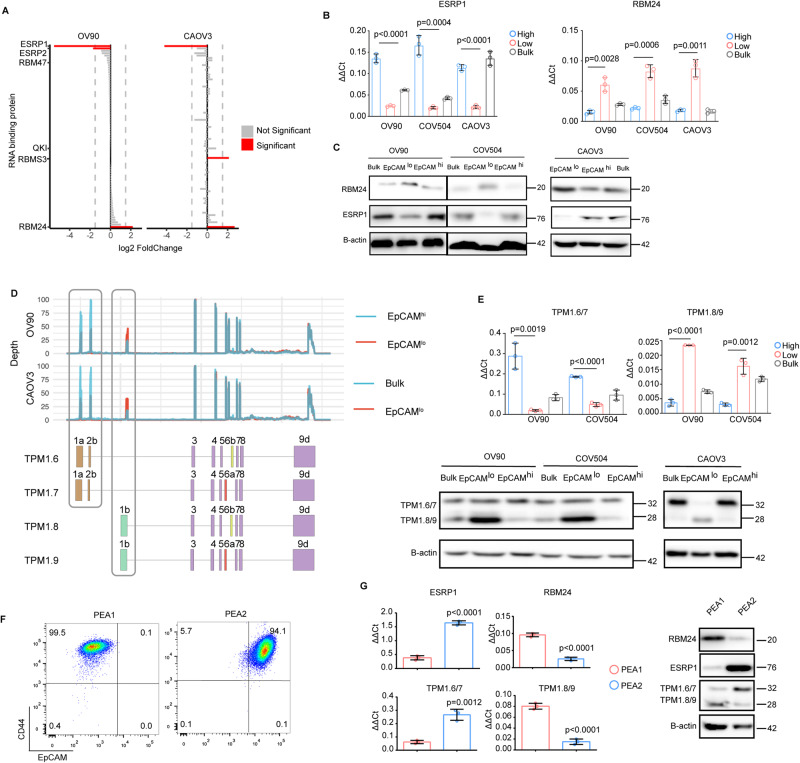
Differential expression of RBPs *ESRP1* and *RBM24* regulates *TPM1* isoforms in HGS ovarian cancer. **A** Fold change analysis of RBPs [original list from reference [14]] differentially expressed between EpCAM^lo^ and EpCAM^hi^ cells in OV90 and CAOV3. Red bar indicates RBPs with significant differential expression (log2-fold change >2, and *P* value < 0.05). The dotted line represents the 1.5 absolute fold change cut-off. **B** RT-qPCR analysis of *ESRP1* and *RBM24* expression in OV90, CAOV3, and COV504 EpCAM^hi/lo^ and bulk cells. GAPDH expression was employed as control (means ± SD, *n* = 3). *P* values are indicated. **C** Western blot analysis of ESRP1 and RBM24 expression in OV90, CAOV3, and COV504 EpCAM^hi/lo^ and bulk cells. β-actin was used as loading control for western blots. **D**. *TPM1* exon peak plots relative to the AS analysis of RNAseq data obtained from OV90 EpCAM^hi/lo^ and CAOV3 bulk/EpCAM^lo^ analysis. Each peak indicates the expression of specific exons; the height of each peak is indicative of the expression level of the specific exons. The exon-intron structure of the corresponding *TPM1* isoforms is depicted below the exon peak plot. Exon 1a and 2b are specific to *Tpm1.6/7* isoforms (brown), while exon 1b is specific to *Tpm1.8/9* (green). Exon 6a (red) is only present in the *Tpm1.7* and *Tpm1.9* isoforms; whereas, exon 6b (yellow) earmarks the *Tpm1.6* and *Tpm1.8* isoforms. With the exception of exon 6a and 6b, exons 3 to 9d are present in all *TPM1* isoforms. **E** RT-qPCR (histogram panels) and western analysis of *TPM1* isoform expression in OV90, CAOV3, and COV504 EpCAM^hi/lo^ and bulk cells. *GAPDH* expression was employed as control. (Means ± SD, *n* = 3). *P* value is indicated. β-actin was used as loading control for western blots. **F** CD44/EpCAM FACS analysis of the ovarian cancer cell lines PEA1 and PEA2. EpCAM/CD44 positive and negative areas were identified using multiple isotype controls. The percentages of cells within each quadrant are indicated. **G** RT-qPCR (left histogram panels) and western (right panel) analysis of ESRP1, RBM24, and *TPM1* isoform expression in PEA1 and PEA2 ovarian cancer cell line; *GAPDH* expression was employed as control. (Means ± SD, *n* = 3). *P* values are indicated. β-actin was used as loading control for western blots.

Recently, we showed that several RNA-binding proteins (RBPs) known to be involved in alternative splicing are differentially expressed between EpCAM^lo^ and EpCAM^hi^ cells in colon cancer cell lines and play significant functional roles in controlling E-to-M and M-to-E transitions and phenotypic plasticity during local dissemination and distant metastasis [17]. By following an analogous approach, we have identified several differentially expressed RBPs likely to play an active role in the regulation of EMT-related alternative isoforms between epithelial- and mesenchymal-like subpopulations of ovarian cancer cells. As shown in Fig. 3A, *ESRP1* and *RBM24* were respectively down- and upregulated in EpCAM^lo^ cells in both cell lines. Moreover, other RBPs (i.e. *ESRP2*, *RBM47*, *RBMS3*, and *QKI*) were differentially expressed in one of the cell lines examined. The EpCAM^lo^-specific up- and downregulation of *RBM24* and *ESRP1*, respectively, was validated by RTqPCR and western analysis of the subpopulations sorted from the OV90, CAOV3 and COV504 cell lines (Fig. 3B, C).

Next, we analyzed differentially spliced genes by MISO (Mixture of Isoforms) [23] and filtered the results by selecting ΔPSI (differential Percentage Spliced In) values > 10% (Supplementary Tables 2 and 3), and by comparing them with the corresponding lists of isoform targets previously found in colon cancer cell lines [17]. As shown in Supplementary Fig. 3 Supplement 1, the large majority of ovarian cancer targets was not found in colon cancer. Among the ovarian-specific targets with a known function in EMT (*n* = 39), diverse cellular components, e.g. extracellular matrix, focal adhesion, and the actin cytoskeleton, and cellular processes, e.g. ECM organization, integrin- and TGFβ-mediated signaling, and cell migration were represented (Supplementary Fig. 3, Supplement 1). In particular, the presence of Tropomyosin 1 (*TPM1*), a member of a broad family of actin-binding proteins, was noteworthy both because of the high ΔPSI values in OV90 and CAOV3 cells and because of its known cellular function, i.e. in the cytoskeleton of non-muscle cells and in the contractile system of striated and smooth muscles [24, 25]. Although a direct causative role between *TPM1* and EMT has not been reported yet, previous studies have shown that TGF-β signaling increases expression of high-molecular weight tropomyosins together with the formation of actin stress fibers thus affecting cell motility and invasion [26, 27].

Notably, the *TPM1* isoform pattern observed in ovarian cancer involved exons 1a/2a, which earmark the *Tpm1.6/7* isoforms upregulated in EpCAM^hi^ cells, and exon 1b, featuring the *Tpm1.8/9* isoforms upregulated in EpCAM^lo^, as validated both by RT-qPCR and western blot analysis (Fig. 3D, E). Of note, the western results relative to *Tpm1.8/9* clearly match the RNA-based analysis, whereas the same is less evident for *Tpm1.6/7* that, at the protein level, seem to undergo more subtle variations. As a further validation of these results, HGS ovarian cancer cell lines exclusively encompassing EpCAM^hi^ (PEA2) or EpCAM^lo^ (PEA1) cells (Fig. 3F), solely expressed the *Tpm1.6/7* and *Tpm1.8/9* isoforms, respectively (Fig. 3G).

**Fig. 4:**
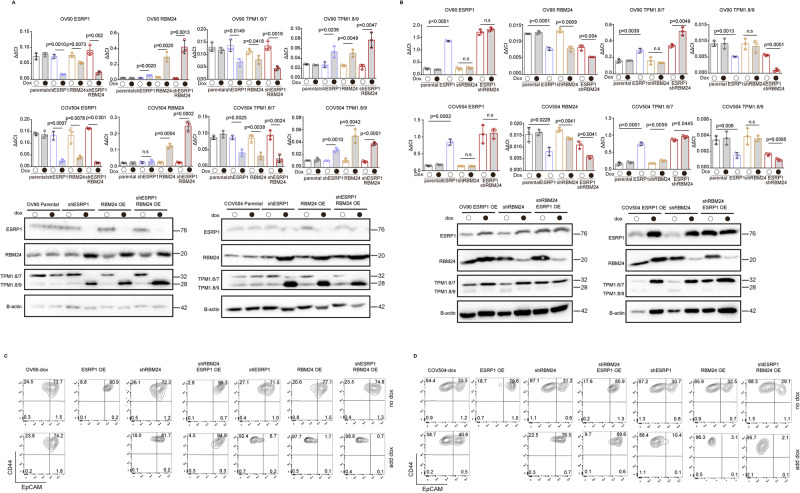
The RBPs *ESRP1* and *RBM24* synergistically regulate *TPM1* isoforms and the relative proportion of EpCAM^hi/lo^ cells. **A** RT-qPCR (histogram panels) and western (lower panel) analysis of ESRP1, RBM24, and *TPM1* isoform expression in *RBM24*-OE (overexpressing) and sh*ESRP1*-KD (knockdown) OV90 and COV504 ovarian cancer cell line; *GAPDH* expression was employed as control (Means ± SD, *n* = 3). β-actin was used as loading control for western blots. **B** RT-qPCR (histogram panels) and western (lower panel) analysis of ESRP1, RBM24, and *TPM1* isoform expression in *ESRP1*-OE (overexpressing) and sh*RBM24*-KD (knockdown) OV90 and COV504 ovarian cancer cell line; *GAPDH* expression was employed as control (Means ± SD, *n* = 3). β-actin was used as loading control for western blots. **C** CD44/EpCAM FACS analysis of *RBM24*-OE/KD and *ESRP1*-OE/KD OV90 cells. Cells were induced with 1 μg/mL doxycycline for 72 h before analysis. The relative percentages of EpCAM^lo^ and EpCAM^hi^ cells are indicated in each quadrant. **D** CD44/EpCAM FACS analysis of *RBM24*-OE/KD and *ESRP1*-OE/KD COV504 cells. Cells were induced with 1 μg/mL doxycycline for 72 h before analysis. The relative percentages of EpCAM^lo^ and EpCAM^hi^ cells are indicated in each quadrant.

In order to establish a cause-effect relationship between the differential expression of *ESRP1* and *RBM24* in EpCAM^lo^ ovarian cancer cells and the observed downstream AS targets using *TPM1* as a model, we performed RBP knockdown and overexpression assays in OV90 and COV504 cell lines. First, as predicted by their unique EpCAM^hi/lo^ distribution, the PEA1 and PEA2 cell lines exclusively express *RBM24* and *ESRP1*, respectively (Fig. 3G, right panel). Accordingly, both *ESRP1* knockdown and *RBM24* ectopic expression in the OV90 and COV504 cell lines resulted in the up- and downregulation of the *Tpm1.8/9* and *Tpm1.6/7* isoforms, respectively, both at the RNA and protein levels (Fig. 4A, Supplementary Fig. 4-Suppl. 1). Vice versa, *RBM24* knockdown and *ESRP1* ectopic expression resulted in the up- and downregulation of the *Tpm1.6/7* and *Tpm1.8/9* isoforms, respectively (Fig. 4B, Supplementary Fig. 4-Suppl. 1). Of note, the observed changes in RBP expression were also accompanied by a pronounced increase of the EpCAM^lo^ subpopulation as shown by FACS analysis (Fig. 4C, Supplementary Fig. 4-Suppl. 2). Concurrent *RBM24* overexpression and *ESRP1* knockdown had significant effects not only on the *TPM1* isoform shift but, more importantly, on the relative percentages of the EpCAM^hi/lo^ subpopulations when compared with the single RBP gain- and loss-of-function assays (Fig. 4, Supplementary Fig. 4-Suppl. 2).

RBPs have been known to play essential roles not only in alternative splicing but also in the co-transcriptional regulation of gene promoters. As the differentially expressed *Tpm1.6/7 and 8/9* isoforms are more likely to result from alternative first exon selection rather than alternative splicing, we performed an in silico analysis of both TPM1a/b promoters using the FANTOM database [28]. No ESRP1 or RBM24 binding motifs were detected. However, the same approach indicated potential interaction with the EMT-TFs SNAI1/2, ZEB1, and RUNX2 (Supplementary Fig. 4-Suppl. 3A, B).

To further study the alternative usage of the TPM1a (Tpm1.6/7) and TPM1b (Tpm1.8/9) promoters, we cloned both promoters into luciferase constructs to assess their transcriptional activity by transient reporter assays. As shown in Supplementary Fig. 4-Suppl. 3C, D, an increase in reporter activity was observed from the TPM1a promoter in the sorted EpCAM^hi^ and in ESRP1-overexpressing OV90 cells. Likewise, increased reporter activity was observed from the TPM1b promoter in the sorted EpCAM^lo^ and in RBM24-overexpressing OV90 and COV504 cells (albeit marginally). Conversely, decreased reporter activity was observed from the TPM1a promoter in sorted OV90 and COV504 EpCAM^hi^ cells upon ESRP1-knockdown. Similarly, decreased reporter activity was observed from the TPM1b promoter in sorted OV90 and COV504 EpCAM^lo^ cells upon RBM24 knockdown (Supplementary Fig. 4-Suppl. 3E-H).

Overall, these results showed that epithelial and quasi-mesenchymal subpopulations of HGS ovarian cancer cells are earmarked by the differential expression of specific RBPs known to be involved in alternative splicing and co-transcriptional regulation of alternative promoters. Accordingly, differential isoform patterns of a subset of EMT-related target genes characterize the EpCAM^lo^ cells. While some of the target gene isoforms are shared with those previously identified in colon cancer, the majority appear to be specific for ovarian cancer. Among these, *TPM1* is of interest in view of both its function as an actin-binding cytoskeletal protein in various cell types [18] and of its previously reported role as a tumor suppressor in breast cancer [29].

### Transcriptional and functional consequences of TPM1 isoforms on quasi-mesenchymal ovarian cancer cells

**Fig. 5:**
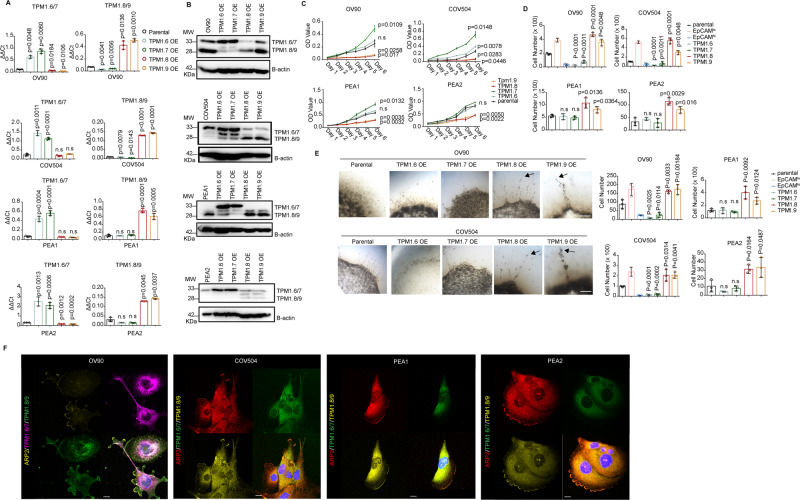
Ectopic expression of *Tpm1.6/7* and *Tpm1.8/9* isoforms results in increased migration and invasion, and decreased cell proliferation. **A** RT-qPCR analysis of OV90, COV504, PEA1, and PEA2 ovarian cancer cell lines transduced to ectopically express the *Tpm1.6/7*-OE and *Tpm1.8/9*-OE isoforms; GAPDH expression was employed as control (Means ± SD, *n* = 3). *P* values are relative to the comparison with the parental cell lines. **B** Western analysis of OV90, COV504, PEA1, and PEA2 ovarian cancer cell lines transduced to ectopically express the *Tpm1.6/7*-OE and *Tpm1.8/9*-OE isoforms. β-actin was employed as loading control. **C** Proliferation assays of OV90, COV504, PEA1, and PEA2 ovarian cancer cell lines transduced to ectopically express the *Tpm1.6/7*-OE and *Tpm1.8/9*-OE isoforms. O.D. values are shown from day 1 to 6 (Means ± SD, *n* = 3). *P* values are relative to the comparison with the parental cell lines. **D** Transwell migration assay of OV90, COV504, PEA1, and PEA2 ovarian cancer cell lines transduced to ectopically express the *Tpm1.6/7*-OE and *Tpm1.8/9*-OE isoforms. 5×10^4^ cells were plated on TC-coated membranes and left O/N. The number of cells that migrated to the lower side of the membrane were counted and plotted (Means ± SD, *n* = 3). *P* values are relative to the comparison with the parental cell lines. **E** Confocal images of OV90 and COV504 parental and *Tpm1.6/7/8/9*-OE cells seeded on collagen layers and incubated for 6 days. As indicated by the arrows, *Tpm1.8/9*-OE cells appear to invade the collagen layer collectively as narrow linear strands with “leader” and “follower” cells. Scale bar: 250 μm. The number of cells invading the collagen was quantified and plotted (Means ± SD, *n* = 3). *P* values are relative to the comparison with the parental cell lines. Plots relative to the PEA1 and PEA2 ovarian cancer cell lines were also calculated (bottom). **F** Immunofluorescence analysis of OV90, COV504, PEA1 and PEA2 parental cells with antibodies directed against ARP2, *Tpm1.6/7* and *Tpm1.8/9*. Nuclei were visualized by DAPI staining of DNA. Scale bar: 5 μm.

To assess the functional relevance of the specific *TPM1* isoforms, their ectopic expression was induced and validated by RT-qPCR and western blot analysis in multiple ovarian cancer cell lines (OV90, COV504, PEA1 and PEA2) (Fig. 5A, B). Cell viability and proliferation assays indicated that *Tpm1.6/7* and *Tpm1.8/9* expression was significantly associated with increased and decreased rates of cell division, respectively (Fig. 5C). Of note, ectopic expression of *Tpm1.8/9* in OV90 and COV504 cells resulted in a complete growth arrest within 6 days. Moreover, trans-well assays clearly showed that *Tpm1.8/9* overexpression resulted in significantly increased migratory and invasive features when compared with *Tpm1.6/7* (Fig. 5D). *Tpm1.8/9*-overexpressing (OE) ovarian cancer cells appeared to invade the collagen layer collectively, as narrow linear strands with “leader” and “follower” cells (Fig. 5E). The latter observation is of interest since in non-muscle cells *Tpm1.8/9* are specifically expressed in lamellipodia, i.e. the membrane protrusions found at the leading edge driven by branched as well as unbranched filaments composed of actin and *Tpm1.8/9* that promote cell motility [30, 31]. IF analysis with *TPM1* isoform-specific antibodies confirmed the co-localization of *Tpm1.8/9* and ARP2, a specific marker for lamellipodia, at the edge of ovarian cancer cells; instead, *Tpm1.6/7* were mainly localized in the cytoplasm (Fig. 5F).

**Fig. 6:**
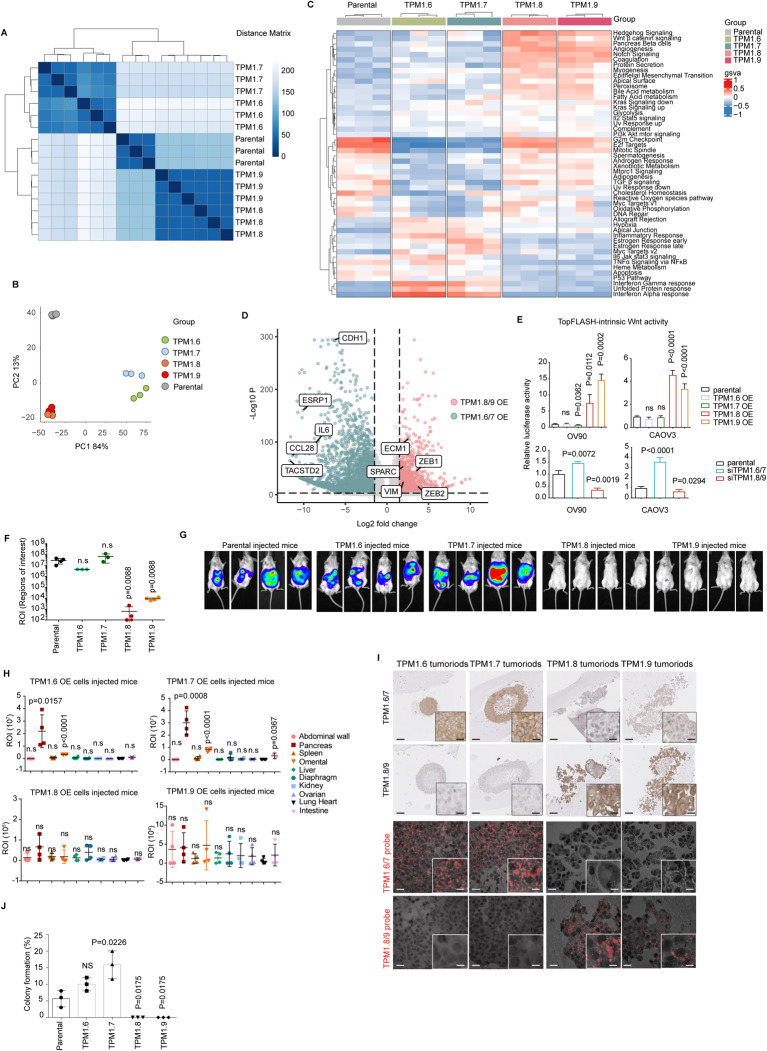
RNAseq analysis revealed *TPM1* isoforms function in Wnt pathway and contribute to metastasis in vivo. **A** Hierarchical clustering of the RNAseq data relative to *Tpm1.6/7/8/9*-OE OV90 cells. Complete-linkage hierarchical clustering was used. **B** Principal component analysis (PCA) of RNAseq profiles from parental and *Tpm1.6/7/8/9*-OE OV90 cells. **C** Hallmarks pathways based on the Gene Set Enrichment Analysis (GSEA) of parental and *Tpm1.6/7/8/9*-OE OV90 cells. The heatmap only includes significantly altered pathways, with NES > 1, and *P* value < 0.05. Complete-linkage hierarchical clustering was used. **D** Volcano plots showing differentially expressed genes between *Tpm1.6/7*-OE (left, green) and *Tpm1.8/9*-OE (pink, right) OV90 cells (abs LFC > 1.5, *P* value < 0.01). **E** TOP-Flash luciferase reporter analysis of Wnt signaling activity in *Tpm1.6/7/8/9*-OE (upper histogram) and upon knockdown by siRNA of *Tpm1.6/7* and *Tpm1.8/9* in OV90 and CAOV3 cells. *P* values are relative to the comparison with the parental cell lines (Means ± SD, *n* = 3-4). **F** Quantification of IVIS bioluminescence signals obtained from NSG mice injected IP with *Tpm1.6/7/8/9*-OE OV90 cells. Recipient animals were sacrificed 5 wk after injection. Four mice were analyzed for each type of transplanted cells. Data are presented as mean values ± SD. Y-axis meaning ROI (regions of interests) are user-defined areas within IVIS optical imaging. **G** Examples of IVIS bioluminescence signals from *Tpm1.6/7/8/9*-OE cell-injected mice at 5 wk after injection. The Spectrum in vivo imaging system was employed. For in vivo imaging purposes, mice were injected IP with D-luciferin (150 mg kg^−1^). **H** IVIS bioluminescence signals relative to specific organs from mice transplanted with the *TPM1* isoform-OE cells. Y-axis meaning ROI (regions of interests) are user-defined areas within IVIS optical imaging. **I** *Tpm1.6/7* and *Tpm1.8/9* IHC (upper panels) and ISH (BaseScope; lower panels) analyses of tumoroids derived from ascetic fluids from mice transplanted with the *TPM1* isoform-OE cells. Scale bars: 100 μm (large panels) and 15 μm (inlets) for IHC; 20 μm and 5 μm (inlets) for ISH. **J** Colony formation assay relative to cells derived from tumoroids obtained from mice transplanted with the *TPM1* isoform-OE cells (Means ± SD, *n* = 3). Y-axis means the percentage of tumoroids single cells form into colonies.

In order to carry out a more comprehensive study of the transcriptional and functional consequences of the ectopic expression of the specific *TPM1* isoforms, RNAseq analysis was carried out on the OV90 overexpressing cells. Unsupervised hierarchical clustering and principal component analyses confirmed the distinct transcriptional identity of the OV90 parental, *Tpm1.6/7*-OE, and *Tpm1.8/9*-OE cells (Fig. 6A, B). Gene set enrichment analysis (GSEA) was then performed to allow the identification of specific signaling pathways and gene ontology functions characteristic of each of the above sample groups. As shown in the heatmap of Fig. 6C, *Tpm1.8/9* overexpression resulted in the pronounced activation of Hedgehog, Wnt/β-catenin, TGF-β, and Notch signaling, i.e. pathways known to be involved in the induction and regulation of EMT. Accordingly, EMT was also found to earmark these cells, as also shown by the distinct expression patterns of several EMT-related genes between *Tpm1.6/7-* and *Tpm1.8/9*-OE samples (Fig. 6D).

To functionally validate the activation of the canonical Wnt/β-catenin signaling pathway, we implemented TopFLASH reporter assays, as previously described [32]. As shown in Fig. 6E, a ~10-fold increase in luciferase activity was observed upon ectopic expression of the individual *Tpm1.8* and *1.9* isoforms. Likewise, Wnt signaling activity was significantly reduced upon *Tpm1.8/9* siRNA-driven knockdown.

Last, to evaluate the in vivo consequences of the ectopic expression of the specific tropomyosin isoforms on their invasive and metastatic capacity, intraperitoneal (IP) injections were performed with *Tpm1.6/7*- and *Tpm1.8/9*-OE cells to model late-stage ovarian cancer with intra-abdominal dissemination and ascites formation. As illustrated in Fig. 6F, G by IVIS imaging and quantification, the results showed that, somewhat surprisingly, IP transplantation of *Tpm1.6/7*-OE cells resulted in increased ascites volumes when compared with *Tpm1.8/9*-OE cells. Accordingly, pancreas and omental metastatic lesions were observed in mice injected IP with *Tpm1.6/7*-OE cells, whereas, no metastases were observed with *Tpm1.8/9*-OE cells (Fig. 6H). These apparently contradictory results are explained by MET repression in ovarian cancer cells constitutively expressing the EMT-inducing *Tpm1.8/9* isoforms. As both E-to-M and M-to-E transitions are necessary for ovarian cancer cell dissemination and metastasis formation, the constitutive induction of EMT in *Tpm1.8/9*-OE cells prevents them from efficiently colonizing the abdominal cavity. As a confirmation of this, “tumoroids” were derived and cultured from the ascites formed in the animals transplanted with ovarian cancer cells overexpressing the *Tpm1.6/7* or *Tpm1.8/9* isoforms. As shown in Fig. 6I, the morphology of these floating cell aggregates derived from *Tpm1.6/7*-OE cells was strikingly structured with multilayered epithelial cells and often with an internal lumen. In sharp contrast, tumoroids from mice transplanted with *Tpm1.8/9*-OE cells lacked any kind of structure and appeared as random aggregates of floating cells. Accordingly, colony formation analysis of single cells from the ascites-derived tumoroids showed that overexpression of the *Tpm1.6/7* results in a substantially increased colonization capacity when compared with *Tpm1.8/9*-OE cells (Fig. 6J).

Overall, these results show that the *TPM1* isoforms confer specific functional features on ovarian cancer cells. In particular *Tpm1.8/9* isoforms are strongly associated with EMT-inducing and inflammatory signaling pathways likely to underlie ‘transcoelomic’ dissemination of ovarian cancer cells and the formation of ascites. However, their ectopic overexpression is also a potential source of artifacts as EMT is equally essential as is MET in the formation of intra-abdominal metastases.

### TPM1.8/9 isoforms confer resistance to taxane- and platinum-based chemotherapy and are expressed in ovarian cancer patients-derived ascites

**Fig. 7:**
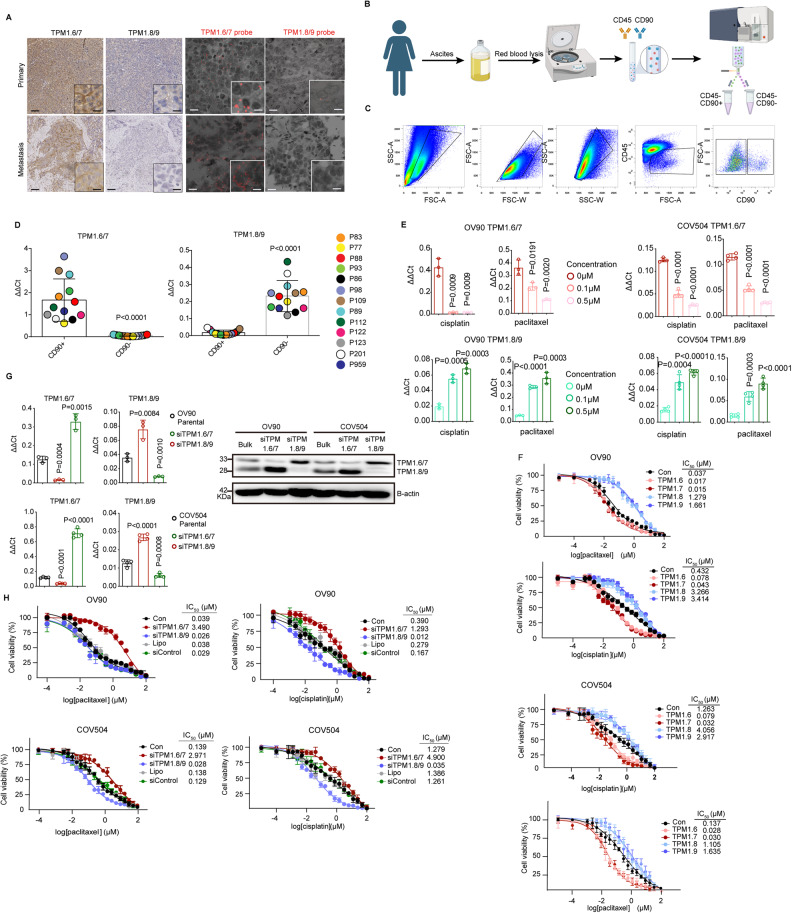
*Tpm1.8/9* isoforms are enriched in malignant ascites from ovarian cancer cells and confer resistance to platinum- and taxane-based therapies. **A** Examples of IHC (left panels) and ISH (BaseScope; right panels) analyses of patient-derived ovarian cancers with antibodies (IHC) and oligonucleotides probes (ISH) specific for the *Tpm1.6/7* and *Tpm1.8/9* isoforms. Ovarian cancer tissues were obtained from a primary tumor and a metastasis (without chemotherapy). Scale bar: 100 μm and 15 μm (inlets) for IHC; 20 μm and 5 μm (inlets) for ISH. **B** Schematic flowchart of the analysis of ascites from late-stage ovarian cancer patients. **C** FACS analysis and sorting strategy of CD45^−^CD90^+^ and CD45^−^CD90^−^ cells from patient-derived ascites. From left to right: FSC-A/SSC–A, FSC-W/FSC-A, SSC-W/SSC-A, FSC-A/CD45^−^ and CD45^-^CD90^+/−^ single cell gates. **D** RT-qPCR analysis of *Tpm1.6/7* and *Tpm1.8/9* expression in sorted CD45^−^CD90^+^ and CD45^−^CD90^−^ cells from patient-derived ascites (*n* = 13) sorted by FACS; GAPDH expression was employed as control (Means ± SD). **E** RT-qPCR analysis of *Tpm1.6/7* and *Tpm1.8/9* expression in OV90 and COV504 cells exposed to cisplatin and paclitaxel cells; GAPDH expression was employed as control (Means ± SD, *n* = 3-4). **F** Dose-response curves relative to *Tpm1.6/7/8/9*-OE cells grown in the presence of different concentrations of paclitaxel and cisplatin (log scale and cell viability on the x and y axis, respectively). IC_50_ values were calculated from biological triplicates to quintuplicates for each experiment (Means ± SD, *n* = 3–5). **G** RT-qPCR (left histogram panels) and western (right) analysis of TPM1 isoform expression in si*Tpm1.6/7* and si*Tpm1.8/9* knockdown OV90 and COV504 cells; GAPDH expression was employed as control (Means ± SD, *n* = 3-4). β-actin was employed as loading control for the western blots. **H** Dose-response curves of si*Tpm1.6/7* and si*Tpm1.8/9* knockdown OV90 and COV504 cells cultured in the presence of different concentrations of paclitaxel (left) and cisplatin (right) concentrations (log scale and cell viability on the x and y axis, respectively). IC_50_ values were calculated from biological triplicates to quintuplicates for each experiment (Means ± SD, *n* = 3–5).

To investigate the role played by *TPM1* alternative isoforms in women suffering from ovarian cancer, patient-derived tumor tissues were examined by immunohistochemistry (IHC) and in situ hybridization (ISH) analyses with isoform-specific antibodies and oligonucleotide probes. Among the high- and low-grade serous tumors analyzed (*n* = 13), *Tpm1.6/7* appeared to be consistently expressed in primary and metastatic lesions (Fig. 7A). In contrast, *Tpm1.8/9* expression was virtually undetectable above background levels, with few cases showing patchy and enhanced staining. Although based on an admittedly limited number of tumors, these observations seem to suggest that, while the *Tpm1.6/7* isoforms are mainly expressed in the bulk of epithelial tumor cells, their *Tpm1.8/*9 counterparts are only rarely observed possibly in association with late and chemo-resistant stages of the disease.

The formation of intra-abdominal ascites predominantly occurs in stage III and IV ovarian cancer patients due to the spreading of tumor cells to the peritoneum and the obstruction of lymphatic vessels. As such, ascites-derived tumor cells may encompass quasi-mesenchymal and chemo-resistant cell types en route to the metastatic colonization of abdominal organs. Hence, we evaluated whether the *Tpm1.8/9* isoforms are transiently expressed in ovarian cancer patient-derived ascites when compared with *Tpm1.6/7*. We collected ascites samples from *n* = 13 patients and sorted the cellular content by FACS according to the [CD45(Lin)^−^/CD90^+^] and [CD45(Lin)^−^/CD90^−^] gates, encompassing immune/stromal and cancer cells, respectively (Fig. 7B, C). Total RNA was then extracted from the sorted cells for RTqPCR analysis of *TPM1* isoform expression. As shown in Fig. 7D, increased *Tpm1.8/9* expression was observed in the ascites-derived cancer cells (CD90^−^) when compared with *Tpm1.6/7*. Among the immune/stromal cells (CD90^+^), *Tpm1.6/7* expression levels were increased when compared with *Tpm1.8/9* (Fig. 7D).

Last, we employed expression profiles from the Cancer Genome Atlas (TCGA) project and the TCGA Splicing Variants Database (TSVdb; http://www.tsvdb.com/) and integrated the clinical follow-up data with the expression of *TPM1* (whole gene) and its isoforms. As shown in Supplementary Fig. 7 Suppl. 1, Kaplan–Meier analysis was borderline significant for the *TPM1* gene and its 1.6/7 isoforms, whereas the p values for *Tpm1.8/9* were both >0.05. The latter is not surprising in view of the very low expression level of these low-molecular weight isoforms in primary ovarian cancer from which the TGCA data are derived.

In view of these results pointing at the specific *Tpm1.8/9* upregulation in ovarian cancer ascites, i.e. at late and recurrent disease stages often in association with chemo-resistance and poor prognosis, we asked whether the same *TPM1* isoforms may confer resistance to the platinum- and taxane-based therapies commonly employed in the clinical management of ovarian cancer. We therefore cultured the OV90, COV504, and CAOV3 parental cell line in the presence of two distinct chemotherapeutic agents, namely cisplatin and paclitaxel, and then analyzed *TPM1* isoform expression in cells surviving the treatment. RTqPCR analysis revealed a dramatic downregulation of *Tpm1.6/7* expression with both agents when compared with the untreated parental cells. In contrast, *Tpm1.8/9* expression appeared to increase in cells surviving both the cisplatin- and paclitaxel-treatment (Fig. 7E). Dose-response curves with OV90, COV504, and CAOV3 cells ectopically expressing the individual isoforms confirmed that *Tpm1.8/9*-OE cells displayed a 40-fold higher resistance (IC_50_ = 3.266/3.414 μM) than *Tpm1.6/7* OE cells (IC_50_ = 0.078/0.043 μM) upon cisplatin treatment in OV90 (Fig. 7F, Supplementary Fig. 7 Suppl. 2). The same was also observed with paclitaxel (IC_50_ = 1.28/1.66 μM for *Tpm1.8/9*-OE; IC_50_ = 0.017/0.015 μM for *Tpm1.6/7*-OE cells). To validate this observation, we developed siRNA assays to selectively downregulate the *Tpm1.6/7* and *Tpm1.8/9* isoforms in parental OV90, COV504, and CAOV3 cells and assess their chemo-resistance. As shown in Fig. 7G, H and Supplementary Fig. 7 Suppl. 2, the specific downregulation of the *Tpm1.8/9* isoforms, validated both at the RNA and protein levels, reduced the cisplatin- and paclitaxel-specific IC_50_, while the opposite was true for the siRNA-driven knockdown of *Tpm1.6/7*. Furthermore, increased resistance to cisplatin and paclitaxel was observed in EpCAM^lo^-sorted cells upon ZEB1 knockdown (by SH) in the OV90 and COV504 cell lines (Supplementary Fig. 7 Suppl. 2).

### TPM1.8/9-specific small molecule inhibitors for ovarian cancer therapy

Given the newly uncovered role played by the *Tpm1.8/9* isoforms in ovarian cancer and the broad spectrum of consequences at the cellular and molecular level that their specific inhibition may exert on cell motility and proliferation, EMT/MET, several oncogenic signaling pathways including Wnt, and therapy resistance, the development of small molecule antagonists may provide novel tools in the clinical management of late-stage ovarian cancer. Differences in the N-and C-termini between tropomyosin isoforms provide an opportunity to develop compounds that preferentially target specific isoforms [33–35]. Compounds that target the C-terminus of TPM3.1 have been shown to inhibit the function of this isoform both in vitro and in vivo by incorporating into the overlap junction between adjacent dimers in actin/Tpm3.1 co-polymers [36, 37]. Compounds targeting the N-terminus show similar activity [38]. Based on the differences in the N-terminal sequences of exon 1b in the *TPM1*, *TPM3*, and *TPM4* genes, compounds were developed that target *Tpm4.2* [39] and *Tpm1.8/1.9* (*European patent application No. 23187348.0*). Virtual screening of compound libraries with the N-terminal model of Tpm1.8/9 was used to identify compounds as potential binders. Targeting of *Tpm1.8/9* by virtual screening identified 6 candidate compounds that were tested for biological activity in human fibroblasts and OV90 cells based on their ability to disperse the target *Tpm1.8/9* away from actin-containing structures in the lamellipodium (Supplementary Fig. 8 Suppl. 1). Two compounds, *Tpm1.8/9*-1 (PubChem CID 6494468) and *Tpm1.8/9*-3 (PubChem CID 18973468) (hereafter for brevity referred to as compounds #1 and #3) were identified with activity in the low micro molar range. Of note, the effective concentration of an inhibitor is influenced by its affinity for the target, metabolism, and other factors, rather than the concentration of the target in the cell. The compounds do not show interaction with the target in cell-free biochemical assays, compatible with either a slow on-rate or fast off-rate resulting in a low affinity interaction with the tropomyosin dimer followed by incorporation into a high affinity binding state in the overlap junction during polymerization. This proposed model is based on previous studies of the incorporation of Tpm3.1 inhibitors into the overlap junction during polymerization.

**Fig. 8:**
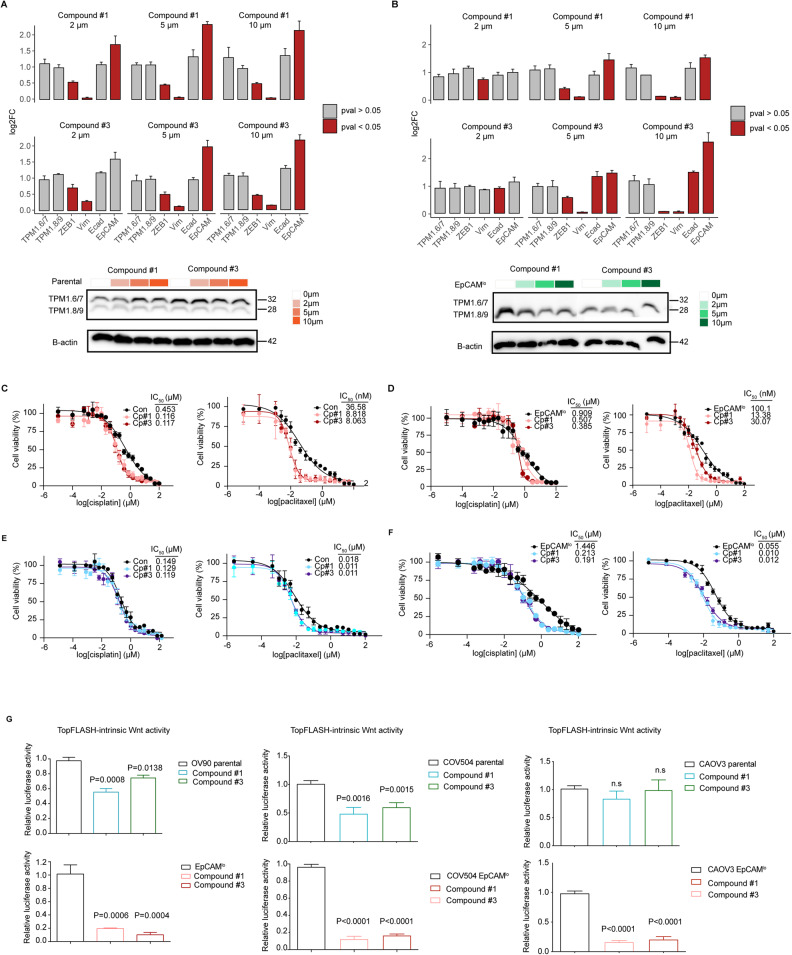
Small molecule inhibitors directed against *Tpm1.8/9* isoforms antagonize their effects on EMT, Wnt signaling, and resistance to chemotherapy. **A** Upper panels: RT-qPCR analysis of *TPM1* isoforms and EMT-related gene expression in OV90 parental cells cultured for 24 h in the presence of compound #1 or #3 at 0, 2, 5, and 10 μM. The values were calculated by normalizing with the untreated cells. *P* values < 0.05 are shown by red bars while gray bars indicate lower values; GAPDH expression was employed as control (Means ± SD, *n* = 3). Lower panels: western analysis of *TPM1* isoform expression in OV90 parental cells cultured for 24 h in the presence of compound #1 or #3 at 0, 2, 5, and 10 μM. Β-actin was used as loading control for western blots. **B** RT-qPCR analysis of *TPM1* isoforms and EMT-related gene expression in OV90 EpCAM^lo^ cells cultured for 24 h in the presence of compound #1 or #3 at 0, 2, 5, and 10 μM. The values were calculated by normalizing with the untreated cells. *P* values < 0.05 are shown by red bars while gray bars indicate lower values; GAPDH expression was employed as control (Means ± SD, *n* = 3). Lower panels: western analysis of *TPM1* isoform expression in OV90 EpCAM^lo^ cells cultured for 24 h in the presence of compound #1 and #3 at 0, 2, 5, and 10 μM. β-actin was used as loading control for western blots. **C** Dose-response curves of parental OV90 cells treated with compound #1 or #3 in the presence of different paclitaxel and cisplatin concentrations. IC_50_ values were calculated from biological triplicates for each experiment (Means ± SD, *n* = 3). **D** Dose-response curves of OV90 EpCAM^lo^ cells treated with compound #1 or #3 in the presence of different paclitaxel and cisplatin concentrations. IC_50_ values were calculated from biological triplicates for each experiment (Means ± SD, *n* = 3). **E** Dose-response curves of parental CAOV3 cells treated with compound #1 or #3 in the presence of different paclitaxel and cisplatin concentrations. IC_50_ values were calculated from biological quadruplicates for each experiment (Means ± SD, *n* = 4). **F** Dose-response curves of CAOV3 EpCAM^lo^ cells treated with compound #1 or #3 in the presence of different paclitaxel and cisplatin concentrations. IC_50_ values were calculated from biological quadruplicates for each experiment (Means ± SD, *n* = 4). **G** TOP-Flash luciferase reporter analysis of Wnt signaling activity in OV90, COV504 and CAOV3 parental (upper panel) and EpCAM^lo^ (lower panel) cells treated with compound #1 or #3 (Means ± SD, *n* = 3–5).

In order to functionally validate the two compounds, we first determined their most effective concentration in the 0 to 10 μM range both on the OV90 parental cell line and on its sorted EpCAM^lo^ subpopulation. RTqPCR and western analysis showed that compounds #1 and #3 do not affect *Tpm1.6/7* or *Tpm1.8/9* expression at either the RNA or protein level (Fig. 8A, B). This is not unexpected since displacement of *Tpm1.8/9* from actin filaments to the soluble pool in cells does not result in *Tpm1.8/9* turnover [40]. However, the expression of EMT-related genes was affected by both compounds: *ZEB1* and *VIM* expression was suppressed, whereas, EpCAM was increased, also in agreement with the expected MET-inducing effects of *Tpm1.8/9* antagonists (Fig. 8A, B). Furthermore, the treated OV90 and CAOV3 bulk and EpCAM^lo^ cells showed a 2–5-fold reduction of the cisplatin- and paclitaxel-specific IC_50_ values when compared with untreated cells (Fig. 8C–F). Last, compounds #1 and #3 dramatically reduced Wnt/β-catenin signaling as shown by TopFLASH reporter assays (Fig. 8G). As expected, the Wnt-inhibiting effects of the compounds are more clearly illustrated by the EpCAM^lo^ cells because of their Wnt- and EMT-hi transcriptional profiles when compared with the parental OV90 and COV504 cell line where a majority of EpCAM^hi^ (Wnt-lo) and EpCAM^lo^ cells coexist.

Overall, these in vitro results suggest the potential for the future development of novel therapeutic strategies for high-grade serous ovarian cancer centered around the *Tpm1.8/9* isoforms.

## Discussion

It is generally accepted that epithelial-mesenchymal plasticity (EMP), i.e. the transient and reversible identity conferred to cancer cells by EMT/MET processes, underlies tumor progression, local invasion, distant metastases, therapy resistance, and immune evasion. As elegantly shown by Cook and Vanderhyden [41, 42], EMT is a highly variable process with a very broad spectrum of upstream signals from the TME, intracellular regulatory mechanisms, and downstream effectors, the identity of which largely depends on the tumor type and its micro- and macro-environment. The same is true when it comes to the nature of the epigenetic mechanisms that underlie EMP [43]. We have recently shown that the differential expression of RNA-binding proteins between the epithelial tumor bulk and subpopulations of quasi-mesenchymal colon cancer cells underlies alterative splicing at a variety of target genes known to play functional roles in EMT, metastasis, and resistance to chemotherapy [17, 21]. Here, we applied a similar strategy toward the identification and functional characterization of genes whose isoform patterns are altered during EMT/MET in ovarian cancer. Comparison of alternatively expressed gene isoforms between epithelial and quasi-mesenchymal colon and ovarian cancer cells revealed few common targets with many ovarian-specific events. This possibly reflect of the distinct modalities of local dissemination and metastatic colonization characteristic of these types of carcinoma. Whereas most colon cancer metastases follow a hematogenous route, unique for ovarian cancer is the ‘transcoelomic’ dissemination of tumor cells and the formation of ascites fluid in the abdominal and pelvic cavity which provide a favorable tumor microenvironment (TME) for the disseminated cancer cells. Nonetheless, previous studies have indicated that EMT does contribute to ovarian cancer progression and to chemotherapy resistance [44]. Hence, even though through distinct cellular and molecular mechanisms, EMT does play a key role in ovarian cancer metastasis and chemo-resistance. Gene ontology analysis of the ovarian cancer specific isoform targets (Supplementary Fig. 3, Supplement 1B-E) revealed an extremely broad spectrum of biological processes, molecular functions, and cellular components likely to collectively contribute to the transition to quasi-mesenchymal ovarian cancer cells capable of local invasion and distant metastatic colonization.

Previously, AS targets likely to contribute to ovarian cancer progression have been reported such as *BCL2L12* [45] and *ECM1* [46]. Here, among the EMT-related isoforms, we selected *TPM1* because of its function in the regulation of cell motility through cytoskeletal modifications [30, 31], and its alleged role as a tumor suppressor and even oncogene in multiple cancer types [47, 48]. *TPM1* isoforms are found in multiple tissues [30, 49]. Our results establish a direct causative relation between the *TPM1.8/9* isoforms and the activation of EMT in ovarian cancer cells. Whether the RBM24 and ESRP1 RBPs, differentially expressed between epithelial and quasi-mesenchymal cells, also play a direct role as co-transcriptional factors of the TPM1a/b promoters, is at present unclear. Analysis of the FANTOM database, while failing to identify known RBP binding sites, did reveal the presence of known EMT-TFs. The results obtained with the luciferase reporter constructs are admittedly inconclusive due to the confounding role of EMT upon ectopic expression or downregulation of the ESRP1 and RBM24 RBPs. Future ChIP or CUT&RUN analyses will elucidate the mechanisms underlying the regulation of Tpm1.6/7 and Tpm1.8/9 isoforms directly and primarily by EMT-TFs or with the RBPs as co-transcriptional factors.

Because of their localization to the lamellipodia and functional role in cell motility, the *TPM1.8/9* isoforms are likely to facilitate dissemination from the primary tumor to the intra-abdominal cavity, as also shown by their enrichment in patient-derived ascites. Allegedly as a consequence of their EMT-inducing capacity, ectopic expression of the low-molecular weight *TPM1* isoforms confers resistance to taxane- and platinum-based chemotherapy. Accordingly, *Tpm1.8/9* are also found to be expressed at very low levels, if any, in primary ovarian cancers and their metastases albeit increased in malignant ascites.

Notwithstanding the above, our attempts to provide in vivo evidence for the metastatic capacity of ovarian cancer cells overexpressing the *Tpm1.8/9* isoforms failed to show any increase when compared with *Tpm1.6/7*. This apparently contradictory result can be explained by the transient and reversible nature of EMT along the multistep events that underlie dissemination and metastasis. As previously proposed by Thomas Brabletz and collaborators [50], while the acquisition of quasi-mesenchymal characteristics is required for local invasion and systemic dissemination, METs are equally essential for the colonization of distant organs. In the OV90 cells overexpressing the EMT-inducing *Tpm1.8/9* isoforms, MET is inhibited thus negatively affecting their metastatic potential. The presence of tumor cells with a strongly compromised colony formation capacity in the malignant ascites from recipient mice transplanted IP with *Tpm1.8/9*-OE cells supports the hypothesis according to which MET suppression in these cells negatively affects their metastatic potential. Another indication of the potential off-target artifacts caused by the non-physiological expression levels of *TPM1* isoforms in these cells became apparent in the analysis of their RNAseq profiles. While several inflammation pathways were upregulated together with *Tpm1.8/9* in the EpCAM^lo^ ovarian cancer cells when compared with their epithelial counterpart, ectopic *Tpm1.6/7* expression resulted in the activation of similar inflammation-related pathways, e.g. IL6/Jak/Stat3, IFN, and TNF, when compared with *Tpm1.8/9* OE cells.

Because of the apparent multifunctional role of *TPM1* isoforms in ovarian cancer malignancy and resistance to therapy, *Tpm1.8/9* forms a potentially relevant therapeutic target. As shown here, the development of small molecule inhibitors will likely prevent EMT and reduce cell motility, and simultaneously inhibit the activation of key signal transduction pathways such as Wnt, known to play a central role in ovarian cancer stemness, EMT, and chemoresistance [51]. Last, combined treatment with conventional taxane- and platinum-based chemotherapies may increase their therapeutic efficacy by antagonizing chemoresistance.

## Materials and methods

### Cell cultures

The human ovarian cancer cell line OV90, obtained from the American Type Culture Collection (ATCC), was cultured in a 1:1 mixture of MCDB 105 medium (M6395; Sigma Aldrich containing 1.5 g/L sodium bicarbonate) and Medium 199 (31150022; Thermo Fisher Scientific containing 2.2 g/L sodium bicarbonate) supplemented with 15% heat inactivated fetal bovine serum (FBS; #16140071, Thermo Fisher Scientific) and 1% Penicillin/Streptomycin (Pen/Strep; penicillin: 100 U/mL, streptomycin: 100 μg/mL; 15140122 Thermo Fisher Scientific). CAOV3 (ATCC), SKOV3 [European Collection of Authenticated Cell Cultures (ECACC) via Sigma], COV504 (ECACC), HEK293T (ATCC) cell lines were cultured in DMEM medium (11965092, Thermo Fisher Scientific) supplemented with 10% heat inactivated FBS, 2 mM L-glutamine (200 mM; 25030081; Thermo Fisher Scientific), and 1% Pen/Strep. PEA1 (ECACC) and PEA2 (ECACC) cell lines were cultured in RPMI 1640 medium (61870036, Thermo Fisher Scientific) with 10% FBS, 1% Pen/Strep, and 2 mM L-glutamine.

The identity of each cell line was confirmed by DNA fingerprinting with microsatellite markers (Amelogenin, CSF1PO, D13S317, D16S539, D5S818, D7S820, THO1, TPOX, vWA, D8S1179, FGA, Penta E, Penta D, D18S51, D3S1358, D21S11) and compared with the analogous data provided by ATCC, EACC, and https://web.expasy.org/cellosaurus/ (data not shown).

### Plasmid transfection and lentiviral transduction

cDNAs encoding *Tpms 1.6, 1.7, 1.8* and *1.9* were excised from the bacterial expression vectors pGEX or pET [from P.W.G. [52]] and cloned into the mammalian expression vector pcDNA3.1(+). Stable transfections of *ESRP1* (Sino Biological plasmid # HG13708-UT) and *Tpm1.6/7/8/9* expression vectors were performed with the FuGENE HD transfection reagent (Promega, E2311) according to the manufacturer’s protocol, and selected with Geneticin (#10131035, Thermo Fisher Scientific).

The inducible pSLIK-RBM24 vector was constructed using pDONR 233-RBM24 as entry plasmid (Horizon, #OHS6084) following Gateway Cloning instructions (11791-020, Thermo Fisher Scientific). The inducible shZEB1 lentiviral vector was obtained as described in our previous study [21]. For all of the above inducible vectors, *ESRP1* (Horizon, V3THS_335722), sh*RBM24* (Horizon, V3SH11240-225117283) lentiviral constructs were packaged by psPAX2 (Addgene # 12260) and pMD2.G (Addgene # 12259) into HEK293T cells. The virus-containing supernatant was collected 24 h after transfection, filtered, and used to infect the OV90 and COV504 cell lines. Selection was applied with 750 ng/mL puromycin (#ant-pr-1, InvivoGen) or 800 μg/mL Geneticin for 1–2 wk. Validation of ectopic expression and knockdown of target genes was conducted 72 h after transfection by qPCR and western blot.

### Luciferase reporter constructs

The promoter regions of TPM1 exons 1a and 1b were used, as reported by Savill et al. [53, 54]. Amplified PCR products were digested with XhoI and SacI, and then ligated into XhoI- and SacI-digested pGL4.10 luciferase reporter plasmid (Promega, Southampton, UK). Constructs were validated by sequencing.

### siRNA transfection

siRNA target sequence for human *Tpm1.6/1.7* was 5’-AAGCTGGAGCTGGCAGAGAAA-3’ (codons 70-76 of *Tpm1.6/1.7* in exon 2b) and for human *Tpm1.8/1.9* was 5’-CGAGAGGAAGCTGAGGGAGAC-3’ (codons 38-44 of *Tpm1.8/1.9* in exon 1b). *Tpm* siRNAs (Horizon Discovery, Waterbeach UK) and control siRNA (#4390843, Thermo Fisher Scientific) were transfected by Lipofectamine RNAiMAX (#13778150, Invitrogen) according to the manufacturer’s instructions. Seventy-two hrs. following siRNA transfection, cells were collected for RNA and protein analysis. To evaluate the effect of gene knockdown on drug resistance, cells were seeded 24 h. after siRNA transfection at a density of 5000 cells/well, followed by 3 days of incubation in the presence of cisplatin (#PHR1624, Sigma-Aldrich) or paclitaxel (#S1150, Selleck Chemicals).

### RT-qPCR and PCR analyses

Total RNA was isolated using the TRIzol reagent (Thermo Fisher Scientific, 15596018) followed by reverse transcription using high-capacity cDNA reverse transcription kit (Life Technologies, 4368814), according to the manufacturer’s instructions. RT-qPCR was performed using the Fast SYBR Green Master Mix (#4368708, Thermo Fisher Scientific) on an Applied Biosystems StepOne Plus Real-Time Thermal Cycling Research device, with three replicates for each analysis. Relative gene expression was determined by normalizing the expression of each target gene to that of *GAPDH*. Results were analyzed using the 2-(ΔΔCt) method. RT-qPCR primers are listed in Supplementary Table 4.

### Western analysis

Cells were lysed in 2X Laemmli buffer (4% SDS, 48% Tris 0.5 M pH6.8, 20% glycerol, 18% H_2_O, bromophenol blue and 10% 1 M DTT), and subjected to SDS-PAGE, followed by transfer onto polyvinylidene fluoride (PVDF) membranes (Bio-Rad). After blocking with 5% milk in TBS-Tween, the membranes were incubated with primary antibodies directed against ESRP1 (1:1000, # PA5-25833, Thermo Fisher Scientific), RBM24 (1:100, #18178-1-AP, Proteintech), *TPM1* (1:1000, #MBS127505, MyBioSource) and β-actin (1:2000, #4970, Cell Signaling). The secondary Ab’s were a goat anti-mouse immunoglobulins/HRP (1:10000, #P0448, DAKO), and a goat anti-rabbit immunoglobulins/HRP (1:10000, #P0161, DAKO). Detection was by the Pierce ECT western blotting substrate (#34578, Thermo Fisher Scientific) using the Amersham AI600 imager (GE Healthcare).

### Flow cytometry analysis and sorting

Single-cell suspensions in PBS supplemented with 1% FBS were incubated with anti-EpCAM-FITC (1:20, #GTX30708, Genetex), and anti-CD44-APC (1:20, #559250, BD Pharmingen) antibodies for 30 min on ice and analyzed on a FACSAria III Cell Sorter (BD Biosciences). CD44^hi^EpCAM^hi^ and CD44^hi^EpCAM^lo^ OV90 and CAOV3 cells were sorted and incubated in a humidified atmosphere at 37 °C with 5% CO_2_ for 3–5 days before RNA or protein were collected as described above. See Fig. 1 for a more detailed protocol and gating specifications.

Patient-derived ascites were first washed 1-2 times with 1$\times$RBC lysis buffer [150 mM NH_4_Cl (#7173-51-5, Sigma Aldrich), 10 mM KHCO_3_ (#298-14-6, Sigma Aldrich), 100 μM EDTA (#60-00-4, Sigma Aldrich)] to remove erythrocytes. The cell pellets were then labeled with anti-CD90 (Brilliant® Violet 421; 1:20, #328122, Clone:5E10, BioLegend), anti-CD45-APC (1:20, #304037, Clone: HI30, BioLegend), and SYTOX^TM^ Red (1:1000, #S34859, Thermo Fisher Scientific) antibodies, and sorted by FACS. RNA from CD45^-^CD90^-^ and CD45^-^CD90^+^ cells was isolated directly after FACS sorting.

### Cell proliferation assays

To analyze cell proliferation rates, 2 ×10^3^ ovarian cancer parental cells and *Tpm1.6/7/8/9*-OE cells were plated into 96-well plates and incubated at 37 °C, 5% CO_2_. After 24 h. (day 1), cells were incubated at 37 °C, 5% CO_2_ for 3 h. in culture medium supplemented with 0.45 mg/mL MTT [(3-(4,5-dimethylthiazol-2-yl)-2,5-diphenyltetrazolium bromide; Sigma-Aldrich]. The 96-well plates were then centrifuged at 1000 rpm for 5 min and the culture medium removed. O.D. reading was performed at 595 nm with a microplate reader (Model 550, Bio-Rad). Background measurements were subtracted from each data point. Experiments were performed in triplicate for each individual cell line.

### Cell migration and invasion assays

Migration assays were conducted using 8 μm pore PET Transwell inserts (#353097, BD Falcon™) and TC-treated multi-well cell culture plates (#353047, BD Falcon™). 5 ×10^4^ cells were seeded in the upper chamber with 100 μL of serum-free culture medium. Culture medium supplemented with 10% FBS was used as a chemoattractant in the lower chamber. After 24 h, cells that migrated to the lower chamber were fixed with 4% PFA (#9713.9010, VWR Chemicals), stained with 0.1% Trypan Blue solution (#15250061, Thermo Fisher Scientific) and counted using a microscope.

The invasion assays were conducted as described here above with the only addition of 200 μL Matrigel mixed with 10–20 μl of a 0.01 M Tris (pH 8.0)/0.7% NaCl solution on top of the Transwell-Clear insert and incubated at 37 °C for 2 h. After having removed the excess liquid, 5 ×10^4^ cells were added to the well in 150 μL serum-free medium. Next, 0.5 mL of culture medium supplemented with 10% FCS was added. After 24 h, the cells that invaded the lower chamber were fixed with 4% PFA and stained with 0.1% Trypan Blue solution. Cells were counted using a microscope.

200 μL collagen mix [7.5% 10 ×PBS, 57% collagen, 0.1% 1 M NaOH, 9.5% H_2_O and 25% culture medium] were plated into scaffolds and incubated at 37 °C for 1 h. 5 ×10^4^ cells were added on top of the solid collagen and incubated at 37 °C. After 1 week, the entire scaffold with cells and collagen was fixed with 4% PFA and embedded in paraffin. 4 μm sections were mounted and counterstained with Hematoxylin. Slides were dehydrated and mounted in Pertex (#00811, Histolab).

### Immunohistochemistry – cultured cells

0.5-1×10^4^ cells were plated into 24-well plates containing glass cover slips coated with 0.2% gelatin. After 6–24 h, culture medium was removed, ice-cold methanol added to each well and plates incubated for 20 min at 4 °C. Cells were washed twice with PBS, and incubated in 0.2% Triton for 20 min with rotation. Cells were blocked in 2% FBS in PBS for 1 h. Primary antibodies for *Tpm1.6/7* (1:200, from P.W.G.), *Tpm1.8/9* (1:200, from P.W.G.), Arp2 (1:200, #ab47654, Abcam) were added and incubated O/N at 4 °C. Cells were washed twice with PBS, and secondary antibodies [goat anti-rat Alexa Fluor 488 conjugate (1:250, #A10528, Life Technologies); goat anti-rabbit Alexa Fluor 546 conjugate (1:250, #A11035, Life Technologies); goat anti-mouse Alexa Fluor 647 conjugate (1:250, #A32728, Life Technologies)] and DAPI (#D1306, Thermo Fisher Scientific) added. Slides were mounted using VECTASHIELD® (#H100010, VECTOR laboratories) and cells imaged using a LSM-700 (Zeiss) with 20$\times$, 40$\times$, 63$\times$ lenses. Images were analyzed using ImageJ.

### Immunochemistry – patient samples

Formalin-fixed, paraffin embedded (FFPE) ovarian cancer patient tissue blocks were obtained from the Department of Pathology, Erasmus Medical Center, Rotterdam. The average fixation time was 1–2 y. 4 μm sections were mounted on slides, dewaxed with Xylene (#28979.294, VWR Chemicals) and hydrated. Antigen retrieval was performed in Tris-EDTA buffer (pH 9.0) using a pressure cooker procedure. Slides were incubated in 3% hydrogen peroxidase (#95321, Sigma Aldrich) at RT for 10 min and blocked with 5% milk (#115363, Millipore) in PBS-Tween (#P1379, Sigma Aldrich) for 30 min. Immunohistochemistry was performed using antibodies directed against *Tpm1.6/7* (1:100), *Tpm1.8/9* (1:100) and mitochondria (1:100, #MAB1273, Sigma Aldrich) followed by the EnVision Plus-HRP system (Dako). Slides were incubated with primary antibodies O/N at 4 °C, washed twice with PBS-Tween and incubated with Rat EnVision+ System-HRP (#P0405, Dako) or Mouse EnVision+ System-HRP (#K4007, Dako) for 30 min. Slides were counterstained with Hematoxylin (#MHS16, Sigma Aldrich).

### In vivo study

Mouse experiments were performed according to the Code of Practice - Animal Experiments in Cancer Research, Netherlands Inspectorate for Health Protection, Commodities and Veterinary Public Health, and the Animal Experiment Committee (DEC). 6–8-week-old NOD.Cg-Prkdc^scid^ Il2rg^tm1Wjl^/SzJ (NSG) female mice were used. Animal randomization was employed. 50 μl PBS containing 1 ×10^5^ OV90 cells overexpressing *Tpm1.6/7/8/9* isoforms was injected IP into each mouse. 150 mg/kg D-luciferin (#L2916, Invitrogen) was injected IP for bioluminescence signal. After 10 min of isoflurane-induced anesthesia (#B506, Zoetis), the bioluminescence of the mouse was measured using the IVIS Spectrum imaging system (Caliper Life Science, Hopkinton, MA) and bioluminescence analyzed using LIVINGIMAGE 4.4 software (Caliper Life Science). Ascites was obtained by syringe, and tumoroids collected and washed with RBC lysis buffer. Mice were sacrificed and tissue fixed in 4% PFA for further analysis.

### Ethics

The Dutch Animal Experimental Committee granted approval for all protocols related to animal research, ensuring adherence to the Code of Practice for Animal Experiments in Cancer Research as outlined by the Netherlands Inspectorate for Health Protections, Commodities, and Veterinary Public Health (The Hague, the Netherlands, 1999).

### Mouse tumoroid culture

Mouse ascites with tumoroids were collected. Tumoroids were washed with RBC lysis buffer 1-2 times, then plated in 24-well ultra-low attachment surface plates (#33019010, Corning). Tumoroids were cultured in a 1:1 mixture of OV90 medium and advanced DMEM/F12 medium (#2322978; Thermo Fisher Scientific) containing 4% B27 (#A1895601, Life Technologies), 2% N-2 supplement (#11520536, Thermo Fisher Scientific) and 0.04% EGF (#PMG8045, Invitrogen).

### BaseScope assay

BaseScope assays were performed following the guidelines from ACD (Advanced Cell Diagnostics, Newark, CA). 4 μm sections were cut onto Superfrost plus slides (#10149870, Thermo Fisher Scientific) and stored O/N at RT. Sections were baked for 1 h at 60 °C before deparaffinizing in xylene and 100% ethanol. Sections were dried for 5 min at 60 °C, incubated in hydrogen peroxide at RT for 10 min, underwent target retrieval for 15 min at 100 °C, and protease treatment for 30 min at 40 °C. BaseScope probes were added and slides incubated in an oven for 2 h at 40 °C before adding reagents AMP1 (30 min at 40 °C), AMP2 (30 min at 40 °C), AMP3 (15 min at 40 °C), AMP4 (30 min at 40 °C), AMP5 (30 min at 40 °C), AMP6 (15 min at RT), AMP7 (30 min at RT) and AMP8 (15 min at RT). Fast Red A and B was added to slides and incubated for 10 min at RT, then counterstained with Gill’s hematoxylin. Slides were dried for 15 min at 60 °C, then mounted in VectaMount permanent mounting medium (H5000, Vector labs). Images were taken using an LSM-700 with 40$\times$ lens.

### Chemoresistance and IC_50_ measurement

Cells were seeded in 96-well plates at 5000 cells/well and left O/N to adhere. 3–5 biological replicates were plated per tested condition. Both cisplatin (#PHR1624, Sigma-Aldrich) and paclitaxel (#S1150, Selleck Chemicals) were dissolved in DMSO (#D2650, Sigma Aldrich). Cells were incubated for 3 days with cisplatin and paclitaxel. After removal of the chemotherapeutic drug, cells were washed with PBS and left to re-grow in standard culture medium for 1 day. Cell viability was assessed using the MTT as described previously [55]. Absolute viability values were converted to percentage viability versus DMSO control treatment, then non-linear fit of log(inhibitor) versus response was performed in GraphPad Prism v7.0 to obtain an IC_50_ values.

### TOP-Flash reporter assay

For the β-catenin/TCF reporter assay (TOP-Flash reporter assay), cells were plated on 48-well dishes. After 48 h, when 70% confluence was reached, cells were transfected by Fugene HD with 125 ng of the TOP-Flash or FOP-Flash reporter constructs together with 25 ng of the Renilla luciferase vector for normalization purposes. Luciferase activity was measured using the Dual-Luciferase Reporter Assay System (#E1910, Promega) 24 h post-transfection. Luminescence was measured using a GloMax Luminometer (#9100-102, Promega).

### Transient transfection and dual-luciferase reporter assay

OV90 and COV504 cells (1 ×10^4^ cells) were transfected with 0.3 pmol of the luciferase reporter plasmid and 0.01 pmol of the Renilla control plasmid using FuGENE6 transfection reagent, following the manufacturer’s instructions. Luciferase activity was measured 24 h post-transfection using the Dual-Luciferase Reporter Assay System (#E1910, Promega). Luminescence was measured with a GloMax Luminometer (#9100-102, Promega).

### Identification of compounds targeting Tpm1.8/1.9

A model of the N-terminus of human Tpm1.8 (identical for Tpm1.9) containing the region of greatest diversity between the four TPM genes (residues 4–16) was constructed. A virtual Screening of a zinc library was performed to identify docking hits to Tpm1.8. The screening protocol consisted of: 1) multiple copy simultaneous search “MCSS” of functional groups such as benzene, pyridine, pyrimidine, pyrazine and phenol around the N-terminus of human Tpm1.8; 2) pharmacophores such as aromatic rings were derived from the distribution of the minima of these fragments; 3) searching the zinc database library (version 2016) based on the pharmacophores, reducing the virtual library of 2553 compounds; 4) docking of these 2553 compounds onto the N-terminus of Tpm1.8 and the best conformations of best overlay with the fragment minima and binding energies to the target were selected. All the calculations were carried out using the software QuCBit [56, 57]. Six compounds were selected and purchased from suppliers. Mouse embryo fibroblasts were exposed to each of the six compounds or vehicle alone for 24 h, fixed and stained for Tpm1.8/1.9 using isoform specific antibodies (Brayford et al.). Control cells show strong enrichment of Tpm1.8/1.9 in the lamellipodium (Brayford et al.). Two of the compounds, Tpm1.8/9-1 (PubChem CID 6494468) and -3 (PubChem CID 18973468), prevented enrichment in the lamellipodium of mouse (not shown) and human fibroblasts at 10 μM (Supplementary Fig. 8 Suppl. 1). They were selected for further studies.

### Alternative splicing analysis

EpCAMhi/lo RNASeq data was obtained from the ovarian cancer cell lines OV90 and CAOV3 and the sequencing reads mapped to GRCh37.p13. genome by STAR [58] (https://www.gencodegenes.org/human/release_19.html). MISO [23] was used to quantify AS events with annotation from https://miso.readthedocs.io/en/fastmiso/index.html#iso-centric. The MISO [23] uses the alternative exon reads and adjacent conservative reads to measure the percentage of transcript isoform with specific exon included, termed Percentage Spliced In (PSI or Ψ). The PSI ranges from 0 (i.e. no isoform includes a specific alternative exon) to 1 (i.e. all of the isoforms detected comprise the alternative exon). GRCh37.p13 was utilized while MISO does not support the GRCh38.p14 assembly.

We removed alternative events with low expression of related transcript isoforms if less than 3 samples in a dataset had more than 10 informative reads to calculate the PSI. Next, we compared the PSI between EpCAM^hi^ and EpCAM^lo^ groups in the OV90 and CAOV3 ovarian cancer cell lines. AS events were defined as differentially spliced events when the difference of mean PSI between two groups (Δpsi; differential Percentage Spliced In) was >10%.

### RNA seq analysis of subpopulations in OV90 and CAOV3

RNA was isolated from sorted populations with Trizol reagent. Libraries were prepared with the TruSeq RNA sample prep kit v2 (Erasmus MC, Biomics). Samples were sequenced with Illumina HiSeq 2000 and adapter sequences were removed with Trimmomatic (v.0.33). Subsequently, the reads were mapped in a two-pass procedure to the human reference genome build hg38 with the RNA-seq aligner STAR (v2.4.2a) [58] using the Homo sapiens GENCODE v23 annotations. Raw counts were imported in DESeq2 (v1.36.0) and normalized with a variance stabilizing transformation (VST) [59]. Differential expressed genes were identified by comparing Epcamlow versus Epcamhigh/bulk samples using absLogFC >1.5 and padj <0.05, and visualized with the ComplexHeatmap [60] package (v2.12.1) after a z-score scaling. Pathways activity was evaluated using gene set enrichment analysis (fgsea v1.22.0) on the hallmark gene sets from the Molecular Signature data base [61, 62]. Complete-linkage hierarchical clustering with split by k-means (k = 2) clustering was used.

### RNA seq analysis of TPM1 OE in OV90

Paired end mRNA sequencing was performed with the DNA Nanoball sequencing (DNBseq) technology till a depth of 25 M reads per sample (BGI Genomics, Shen Zhen). Adapter trimming and quality filtering was performed using the SOAPnuke pipeline (BGI Genomics). Clean FASTQ files were aligned to the GRCh37 reference genome with RSEM (v1.3.3) [63] using the STAR aligner (v2.7.9a) [58]. Gene level data was imported with tximport [64] (v1.24.0) and downstream analysis was performed using DESeq2 (v1.36.0) [59]. Counts were normalized with a variance stabilizing transformation (VST). Gene set activity was evaluated with a gene set variation analysis (GSVA, v1.44.5) [65] using the Hallmark gene set from the Molecular Signature database [61] and visualized with the ComplexHeatmap [60] package (v2.12.1). Principal component analysis was computed using the top 500 genes with highest row variance. Differential expression analysis was performed by comparing the TPM1.6/7 samples with the TPM1.8/9 samples and results were displayed with a volcano plot using EnhancedVolcano (v1.14.0). Complete-linkage hierarchical clustering was used.

### scRNAseq analysis of ovarium cancer cells

Publicly available data from Vázquez-García et al. were retrieved from CellxGene portal [22]. Downstream analysis was performed in Seurat (v4.3.0) [66]. An EpCAM^lo^ signature was evaluated with AddModuleScore based on the previously identified upregulated gene list (*N* = 38). A threshold (>0.1) was used to annotate cells with the highest association to the EpCAM^lo^ signature (“low-like cells”, 4% of cancer cells). Next, low-like cells were visualized on the integrated UMAP embedding from Vázquez-García et al. and pathway activity of clusters encompassing low-like cells were visualized with ComplexHeatmap [60]. After approval of a data transfer agreement, FASTQ files from Izar et al. [67] were downloaded from the TerraBio repository and processed with RSEM using the STAR aligner to the hg19 human reference genome with isoform annotation from UCSC [58, 63]. Files were imported with tximport [64] (v1.24.0) and cells were selected that contained at least 500 different genes (nFeature_RNA > 500). Cells were clustered (kmeans, k = 4) according to the percentage of their respective TPM1 isoform expression and subsequent analysis was performed in Seurat (v4.3.0) [66], where TPM1.7 expressing cells were compared to TPM1.9 with FindMarkers. A gene set enrichment analysis was performed using the Hallmark gene set and pathways were filtered according to similar activity in the OV90 cell line and patient data. Complete-linkage hierarchical clustering was used.

### Survival analysis of TPM1 in TCGA OVCA

RSEM processed data from the TCGA cohort was downloaded from the tsvDB [68]. Data was log2 transformed and survival analysis was performed with the survival package. Survival curves were generated with the survminer package for the whole TPM1 gene, TPM1.7 (isoform_uc002alk) and TPM1.9 (isoform_uc002alt) based on clinical data on overall survival [69].

### Statistical analysis

For statistical comparison, we performed unpaired *t test*. Statistical analyses were performed using Prism 7 software (GraphPad). Data with statistical significance are as indicated. Information on replicates, independent experiments and statistical test can be found in the Fig. Legends. Analysis tools were run with all parameters as default unless otherwise stated.

### Supplementary information

Original data for Blots

Supplementary Figure Legends

Fig.3-Suppl1

Fig.4-Suppl1

Fig.4-Suppl2

Fig.4-Suppl3

Fig.6-Suppl1

Fig.7-Suppl1

Fig.7-Suppl2

Fig.8-Suppl1

Fig.8-Suppl2

reproducibility checklist

Supplementary Table1

Supplementary Table2

Supplementary Table3

Supplementary Table4

## Data Availability

RNA sequencing data has been deposited to the Gene Expression Omnibus (GEO) and can be accessed using the following identifiers: GSE192920 (subpopulations in OV90 and CAOV3), GSE231560 (TPM1 OE in OV90). Other single cell RNA sequencing data used in this study are publicly available and can be accessed from GEO for the SmartSeq2 data [GSE146026 [67]] and from Synapse for the 10X Genomics data [syn25569736 [22]].

## References

[1] Jayson GC, Kohn EC, Kitchener HC, Ledermann JA (2014). Ovarian cancer. Lancet.

[2] Siegel RL, Miller KD, Jemal A (2018). Cancer statistics, 2018. CA Cancer J Clin.

[3] Bast RC, Hennessy B, Mills GB (2009). The biology of ovarian cancer: new opportunities for translation. Nat Rev Cancer.

[4] Kurman RJ, Shih IeM (2008). Pathogenesis of ovarian cancer: lessons from morphology and molecular biology and their clinical implications. Int J Gynecol Pathol.

[5] Bowtell DD, Böhm S, Ahmed AA, Aspuria P-J, Bast RC, Beral V (2015). Rethinking ovarian cancer II: reducing mortality from high-grade serous ovarian cancer. Nat Rev Cancer.

[6] Naora H, Montell DJ (2005). Ovarian cancer metastasis: integrating insights from disparate model organisms. Nat Rev Cancer.

[7] Kipps E, Tan DSP, Kaye SB (2013). Meeting the challenge of ascites in ovarian cancer: new avenues for therapy and research. Nat Rev Cancer.

[8] Lengyel E (2010). Ovarian cancer development and metastasis. Am J Pathol.

[9] Samatov TR, Tonevitsky AG, Schumacher U (2013). Epithelial-mesenchymal transition: focus on metastatic cascade, alternative splicing, non-coding RNAs and modulating compounds. Mol Cancer.

[10] Blencowe BJ (2006). Alternative splicing: new insights from global analyses. Cell.

[11] Bechara EG, Sebestyen E, Bernardis I, Eyras E, Valcarcel J (2013). RBM5, 6, and 10 differentially regulate NUMB alternative splicing to control cancer cell proliferation. Mol Cell.

[12] Lagier-Tourenne C, Polymenidou M, Hutt KR, Vu AQ, Baughn M, Huelga SC (2012). Divergent roles of ALS-linked proteins FUS/TLS and TDP-43 intersect in processing long pre-mRNAs. Nat Neurosci.

[13] Ohno G, Ono K, Togo M, Watanabe Y, Ono S, Hagiwara M (2012). Muscle-specific splicing factors ASD-2 and SUP-12 cooperatively switch alternative pre-mRNA processing patterns of the ADF/cofilin gene in Caenorhabditis elegans. PLoS Genet.

[14] Fu XD, Ares M (2014). Context-dependent control of alternative splicing by RNA-binding proteins. Nat Rev Genet.

[15] Oltean S, Bates DO (2014). Hallmarks of alternative splicing in cancer. Oncogene.

[16] Wang S, Sun Z, Lei Z, Zhang HT (2022). RNA-binding proteins and cancer metastasis. Semin Cancer Biol.

[17] Xu T, Verhagen M, Joosten R, Sun W, Sacchetti A, Munoz Sagredo L (2022). Alternative splicing downstream of EMT enhances phenotypic plasticity and malignant behavior in colon cancer. Elife.

[18] Gunning PW, Hardeman EC (2017). Tropomyosins. Curr Biol.

[19] Mlakar V, Berginc G, Volavsek M, Stor Z, Rems M, Glavac D (2009). Presence of activating KRAS mutations correlates significantly with expression of tumour suppressor genes DCN and TPM1 in colorectal cancer. BMC Cancer.

[20] Zhu S, Si ML, Wu H, Mo YY (2007). MicroRNA-21 targets the tumor suppressor gene tropomyosin 1 (TPM1). J Biol Chem.

[21] Sacchetti, A. et al. Phenotypic plasticity underlies local invasion and distant metastasis in colon cancer. Elife 10, 10.7554/eLife.61461 (2021) 10.7554/eLife.61461 PMC8192123 34036938

[22] Vazquez-Garcia I, Uhlitz F, Ceglia N, Lim JLP, Wu M, Mohibullah N (2022). Ovarian cancer mutational processes drive site-specific immune evasion. Nature.

[23] Katz Y, Wang ET, Airoldi EM, Burge CB (2010). Analysis and design of RNA sequencing experiments for identifying isoform regulation. Nat Methods.

[24] Pittenger MF, Kazzaz JA, Helfman DM (1994). Functional properties of non-muscle tropomyosin isoforms. Curr Opin Cell Biol.

[25] Groger H, Callaerts P, Gehring WJ, Schmid V (1999). Gene duplication and recruitment of a specific tropomyosin into striated muscle cells in the jellyfish Podocoryne carnea. J Exp Zool.

[26] Zheng Q, Safina A, Bakin AV (2008). Role of high-molecular weight tropomyosins in TGF-beta-mediated control of cell motility. Int J Cancer.

[27] Bakin AV, Safina A, Rinehart C, Daroqui C, Darbary H, Helfman DM (2004). A critical role of tropomyosins in TGF-beta regulation of the actin cytoskeleton and cell motility in epithelial cells. Mol Biol Cell.

[28] Carninci P, Kasukawa T, Katayama S, Gough J, Frith MC, Maeda N (2005). The transcriptional landscape of the mammalian genome. Science.

[29] Bharadwaj S, Prasad GL (2002). Tropomyosin-1, a novel suppressor of cellular transformation is downregulated by promoter methylation in cancer cells. Cancer Lett.

[30] Brayford S, Bryce NS, Schevzov G, Haynes EM, Bear JE, Hardeman EC (2016). Tropomyosin Promotes Lamellipodial Persistence by Collaborating with Arp2/3 at the Leading Edge. Curr Biol.

[31] Cagigas ML, Bryce NS, Ariotti N, Brayford S, Gunning PW, Hardeman EC (2022). Correlative cryo-ET identifies actin/tropomyosin filaments that mediate cell-substrate adhesion in cancer cells and mechanosensitivity of cell proliferation. Nat Mater.

[32] Korinek V, Barker N, Morin PJ, van Wichen D, de Weger R, Kinzler KW (1997). Constitutive transcriptional activation by a beta-catenin-Tcf complex in APC-/- colon carcinoma. Science.

[33] Stehn JR, Schevzov G, O’Neill GM, Gunning PW (2006). Specialisation of the tropomyosin composition of actin filaments provides new potential targets for chemotherapy. Curr Cancer Drug Targets.

[34] Stehn JR, Haass NK, Bonello T, Desouza M, Kottyan G, Treutlein H (2013). A novel class of anticancer compounds targets the actin cytoskeleton in tumor cells. Cancer Res.

[35] Currier MA, Stehn JR, Swain A, Chen D, Hook J, Eiffe E (2017). Identification of Cancer-Targeted Tropomyosin Inhibitors and Their Synergy with Microtubule Drugs. Mol Cancer Ther.

[36] Bonello TT, Janco M, Hook J, Byun A, Appaduray M, Dedova I (2016). A small molecule inhibitor of tropomyosin dissociates actin binding from tropomyosin-directed regulation of actin dynamics. Sci Rep.

[37] Janco M, Rynkiewicz MJ, Li L, Hook J, Eiffe E, Ghosh A (2019). Molecular integration of the anti-tropomyosin compound ATM-3507 into the coiled coil overlap region of the cancer-associated Tpm3.1. Sci Rep.

[38] Hardeman E, Gunning P, E. E, inventors; TroBio Therapeutics, Pty Ltd., assignee. Sulfonamide compounds and the use thereof in the treatment of cancer. 2021. Patent: WO2021072487

[39] Hardeman E, Gunning P, Eiffe E, inventors; TroBio Therapeutics, Pty Ltd, assignee. Substituted indole compounds and the use thereof. 2022. Patent: WO2020037079A1

[40] Meiring JCM, Bryce NS, Wang Y, Taft MH, Manstein DJ, Liu Lau S (2018). Co-polymers of Actin and Tropomyosin Account for a Major Fraction of the Human Actin Cytoskeleton. Curr Biol.

[41] Cook DP, Vanderhyden BC (2020). Context specificity of the EMT transcriptional response. Nat Commun.

[42] Cook DP, Vanderhyden BC (2022). Transcriptional census of epithelial-mesenchymal plasticity in cancer. Sci Adv.

[43] Skrypek N, Goossens S, De Smedt E, Vandamme N, Berx G (2017). Epithelial-to-Mesenchymal Transition: Epigenetic Reprogramming Driving Cellular Plasticity. Trends Genet.

[44] Loret N, Denys H, Tummers P, Berx G (2019). The Role of Epithelial-to-Mesenchymal Plasticity in Ovarian Cancer Progression and Therapy Resistance. Cancers.

[45] Wang Z, Wang S, Qin J, Zhang X, Lu G, Liu H (2022). Splicing factor BUD31 promotes ovarian cancer progression through sustaining the expression of anti-apoptotic BCL2L12. Nat Commun.

[46] Yin H, Wang J, Li H, Yu Y, Wang X, Lu L (2021). Extracellular matrix protein-1 secretory isoform promotes ovarian cancer through increasing alternative mRNA splicing and stemness. Nat Commun.

[47] Varga AE, Stourman NV, Zheng Q, Safina AF, Quan L, Li X (2005). Silencing of the Tropomyosin-1 gene by DNA methylation alters tumor suppressor function of TGF-beta. Oncogene.

[48] Pan H, Gu L, Liu B, Li Y, Wang Y, Bai X (2017). Tropomyosin-1 acts as a potential tumor suppressor in human oral squamous cell carcinoma. PloS one.

[49] Hardeman EC, Bryce NS, Gunning PW (2020). Impact of the actin cytoskeleton on cell development and function mediated via tropomyosin isoforms. Semin Cell Dev Biol.

[50] Brabletz T, Jung A, Spaderna S, Hlubek F, Kirchner T (2005). Opinion: migrating cancer stem cells - an integrated concept of malignant tumour progression. Nat Rev Cancer.

[51] Teeuwssen M, Fodde R (2019). Wnt Signaling in Ovarian Cancer Stemness, EMT, and Therapy Resistance. J Clin Med.

[52] Schevzov G, Whittaker SP, Fath T, Lin JJ, Gunning PW (2011). Tropomyosin isoforms and reagents. Bioarchitecture.

[53] Savill SA, Leitch HF, Daly AK, Harvey JN, Thomas TH (2010). Polymorphisms in the tropomyosin TPM1 short isoform promoter alter gene expression and are associated with increased risk of metabolic syndrome. Am J Hypertens.

[54] Savill SA, Leitch HF, Harvey JN, Thomas TH (2012). Functional structure of the promoter regions for the predominant low molecular weight isoforms of tropomyosin in human kidney cells. J Cell Biochem.

[55] Sacchetti A, Teeuwssen M, Verhagen M, Joosten R, Xu T, Stabile R (2021). Phenotypic plasticity underlies local invasion and distant metastasis in colon cancer. Elife.

[56] Zeng J, Treutlein HR (1999). A method for computational combinatorial peptide design of inhibitors of Ras protein. Protein Eng.

[57] Zeng J (2000). Mini-review: computational structure-based design of inhibitors that target protein surfaces. Comb Chem High Throughput Screen.

[58] Dobin A, Davis CA, Schlesinger F, Drenkow J, Zaleski C, Jha S (2013). STAR: ultrafast universal RNA-seq aligner. Bioinformatics.

[59] Love MI, Huber W, Anders S (2014). Moderated estimation of fold change and dispersion for RNA-seq data with DESeq2. Genome Biol.

[60] Gu Z, Hubschmann D (2022). Make Interactive Complex Heatmaps in R. Bioinformatics.

[61] Subramanian A, Tamayo P, Mootha VK, Mukherjee S, Ebert BL, Gillette MA (2005). Gene set enrichment analysis: a knowledge-based approach for interpreting genome-wide expression profiles. Proc Natl Acad Sci USA.

[62] Gennady Korotkevich, V. S., Nikolay Budin, Boris Shpak, Maxim N. Artyomov, Alexey Sergushichev. Fast gene set enrichment analysis. 10.1101/060012 (2021).

[63] Li B, Dewey CN (2011). RSEM: accurate transcript quantification from RNA-Seq data with or without a reference genome. BMC Bioinforma.

[64] Soneson C, Love MI, Robinson MD (2015). Differential analyses for RNA-seq: transcript-level estimates improve gene-level inferences. F1000Res.

[65] Hanzelmann S, Castelo R, Guinney J (2013). GSVA: gene set variation analysis for microarray and RNA-seq data. BMC Bioinforma.

[66] Hao Y, Hao S, Andersen-Nissen E, Mauck WM, Zheng S, Butler A (2021). Integrated analysis of multimodal single-cell data. Cell.

[67] Izar B, Tirosh I, Stover EH, Wakiro I, Cuoco MS, Alter I (2020). A single-cell landscape of high-grade serous ovarian cancer. Nat Med.

[68] Sun W, Duan T, Ye P, Chen K, Zhang G, Lai M (2018). TSVdb: a web-tool for TCGA splicing variants analysis. BMC Genomics.

[69] Therneau TM, Grambsch PM. Modeling Survival Data: Extending the Cox Model. 10.1007/978-1-4757-3294-8 (2001).

