# Supplementary material for: Tropomyosin1 isoforms underlie epithelial to mesenchymal plasticity, metastatic dissemination, and resistance to chemotherapy in high-grade serous ovarian cancer
Source: Cell Death Differ. 2024 Feb 16;31(3):360–77. doi: 10.1038/s41418-024-01267-9 (PMC10923901; doi:10.1038/s41418-024-01267-9)

Figure 3C RBM24 in OV90

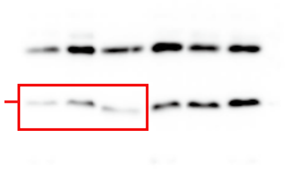

Figure 3C ESRP1 in COV504

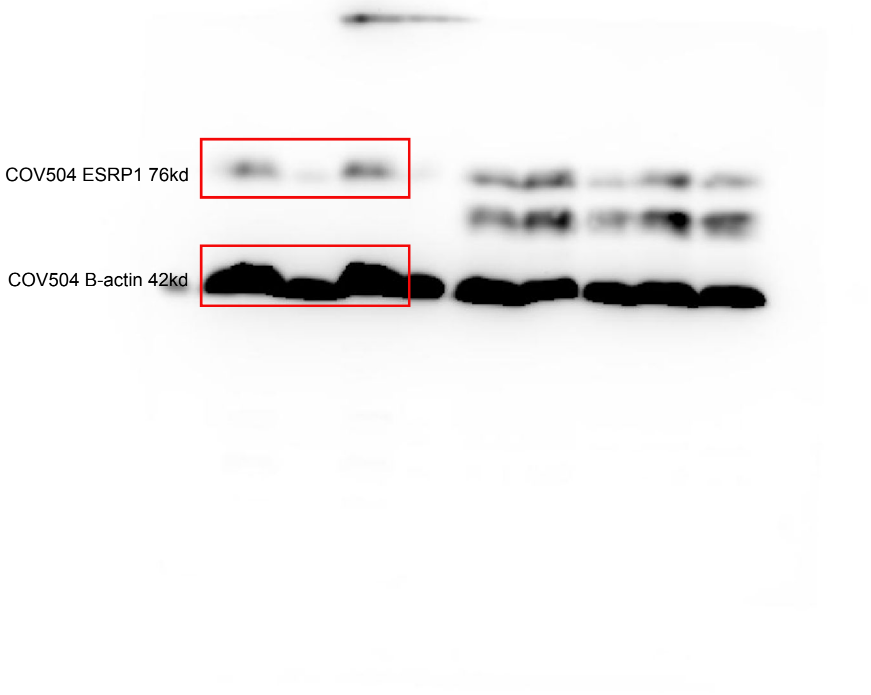

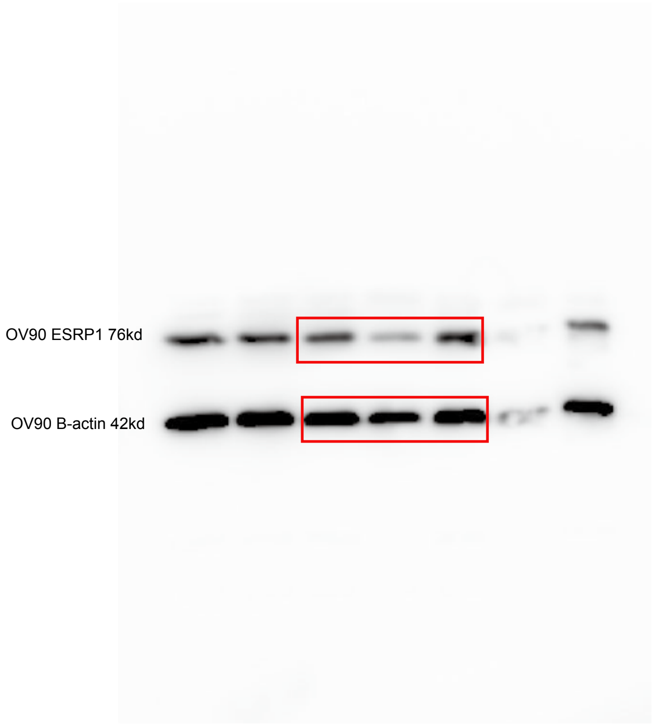
OV90 ESRP1 76kd
OV90 B-actin 42kd

Figure 3C ESRP1 in CAOV3

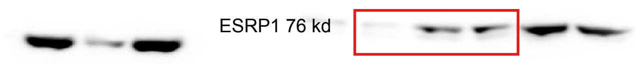

Figure 3C RBM24 in CAOV3

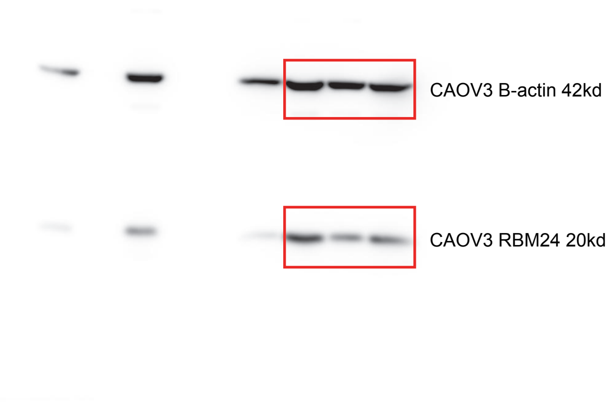

Figure 3C RBM24 in COV504

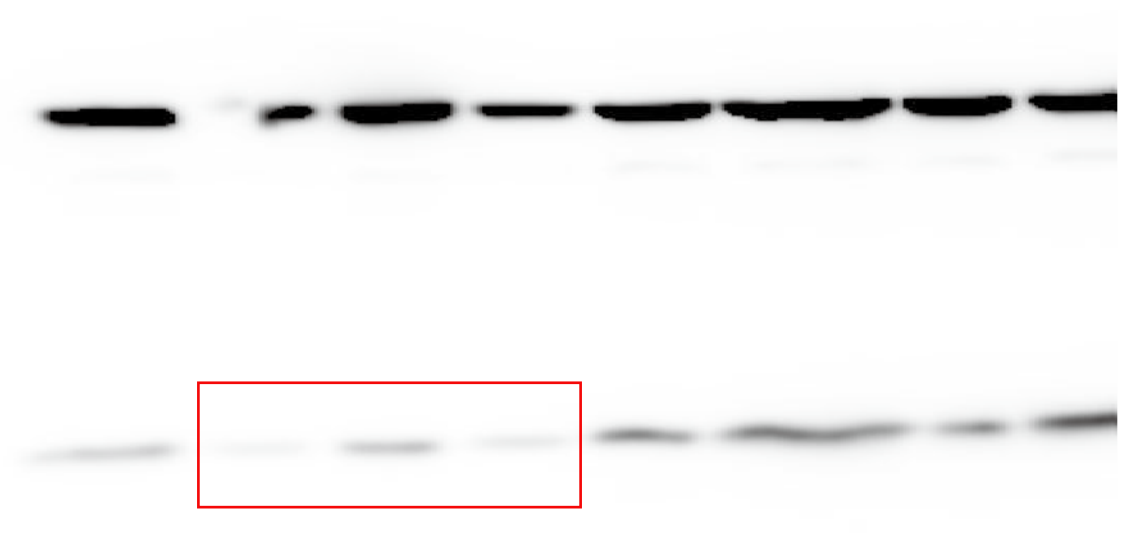

COV504 RBM24 20kd

Figure 3E TPM1 in CAOV3

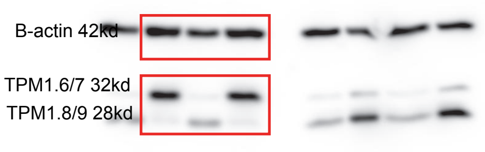

Figure 3E TPM1 in OV90 and COV504

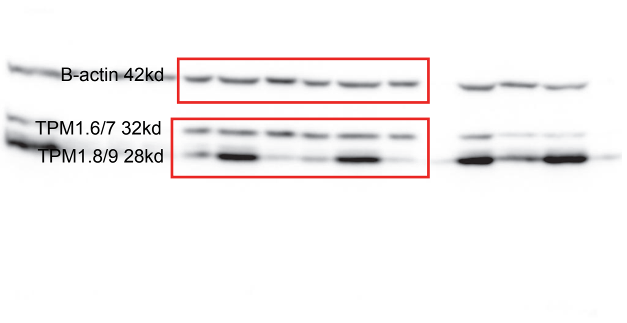

Figure 3G ESRP1 in PEA1 and PEA2

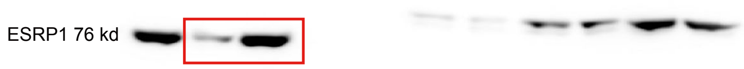

Figure 3G RBM24 in PEA1 and PEA2

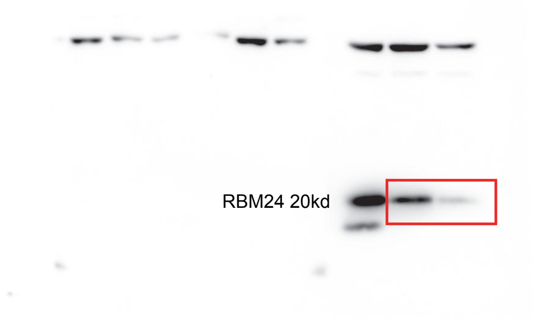

Figure 3G TPM1 in PEA1 and PEA2

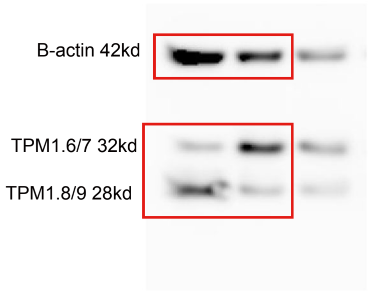

Figure 4A left ESRP1 in OV90

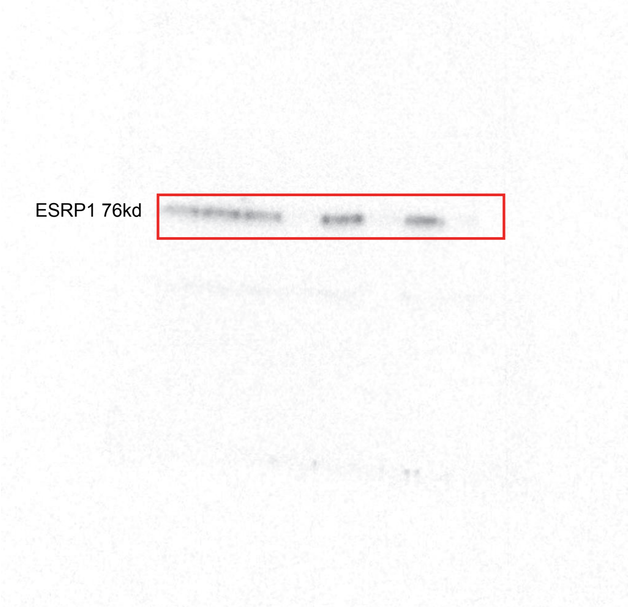

Figure 4A left RBM24 in OV90

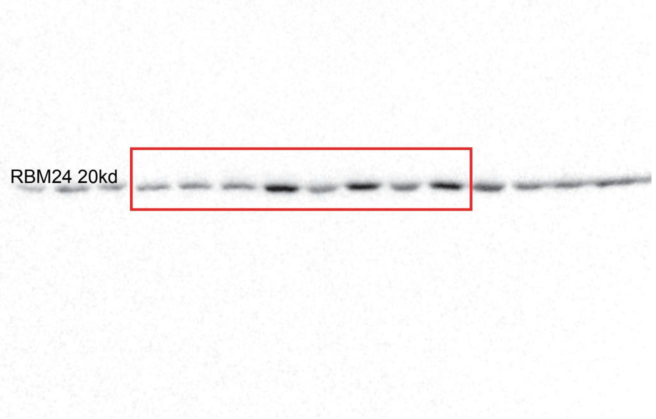

Figure 4A left TPM1 in OV90

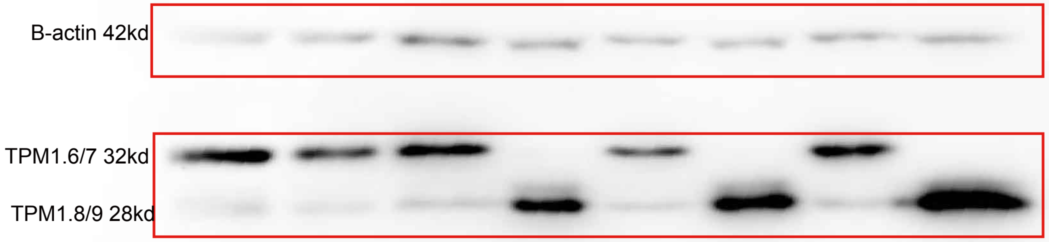

Figure 4A right TPM1 in COV504

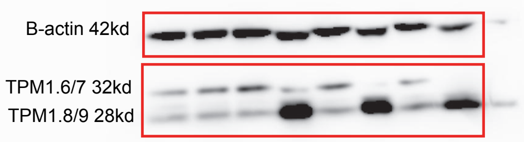

Figure 4A right ESRP1 in COV504

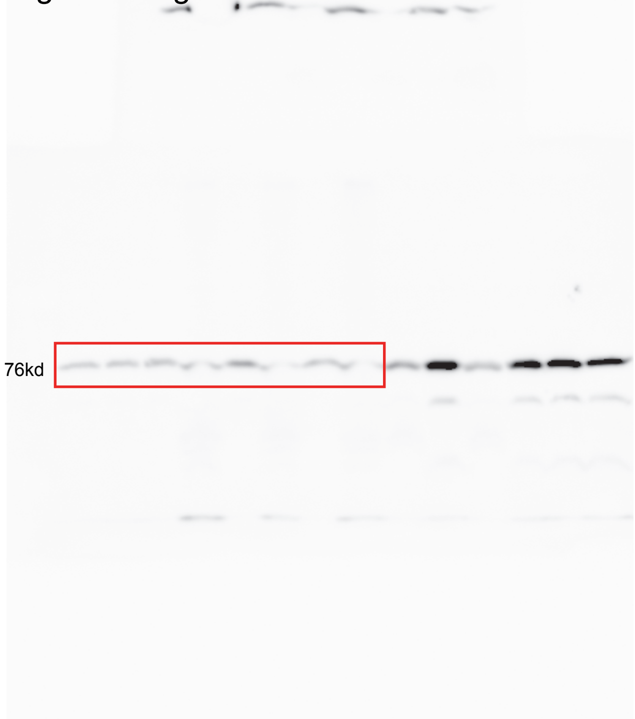

Figure 4A right RBM24 in COV504

RBM24 20kd

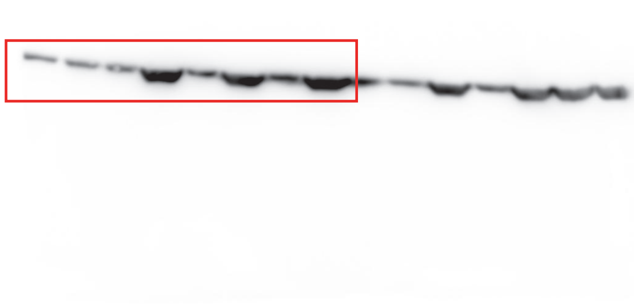

Figure 4B left ESRP1 in OV90

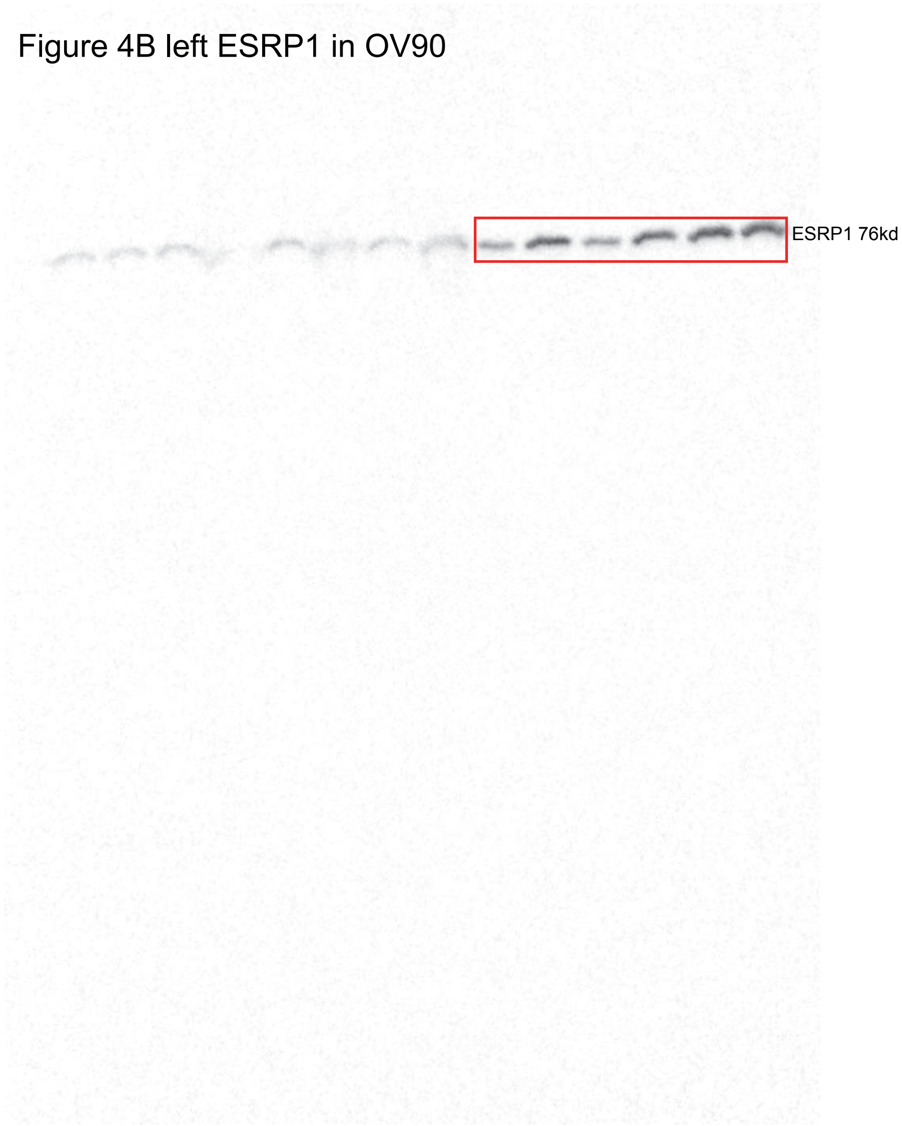

Figure 4B left RBM24 in OV90

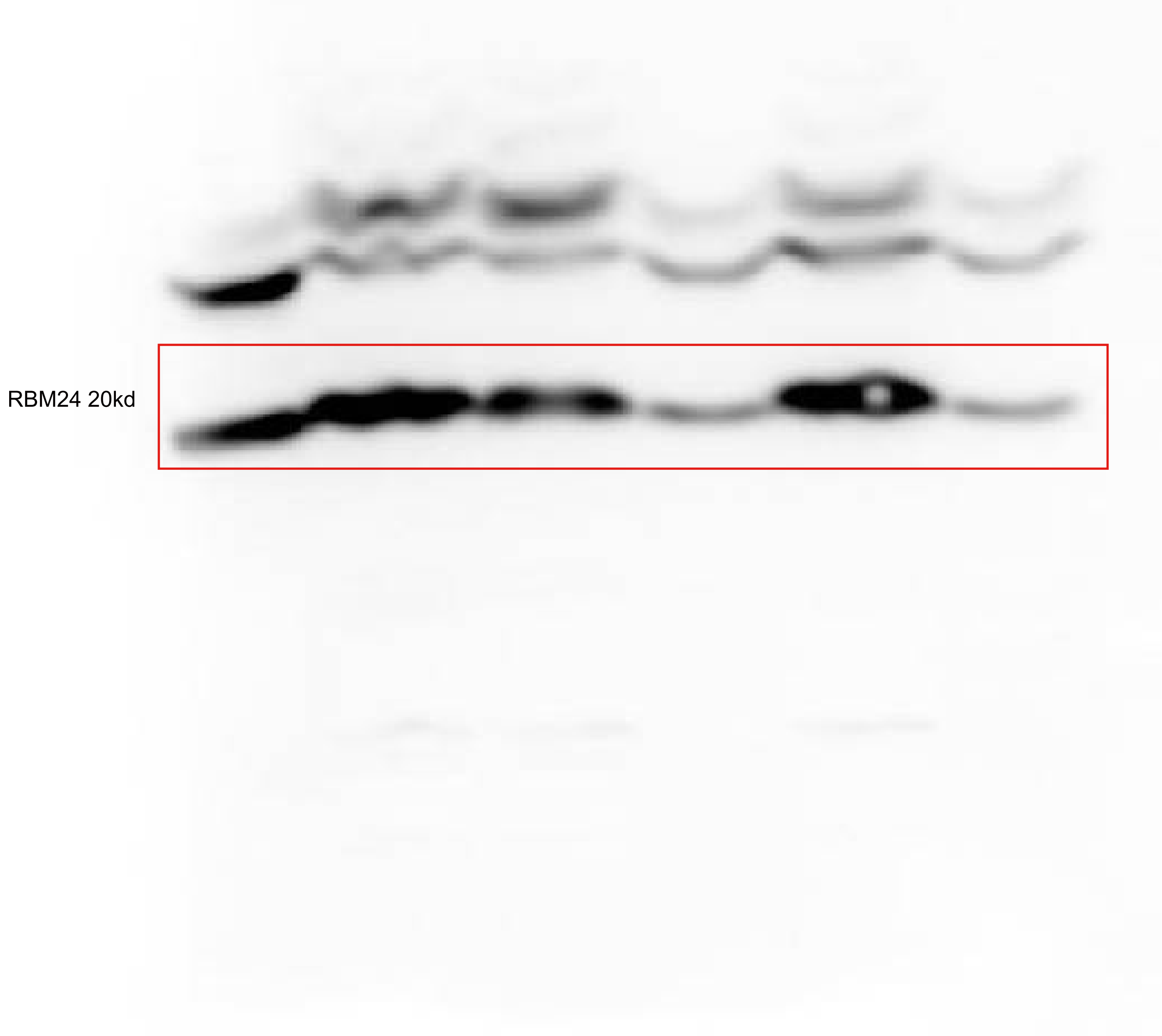

Figure 4B left TPM1 B-actin in OV90

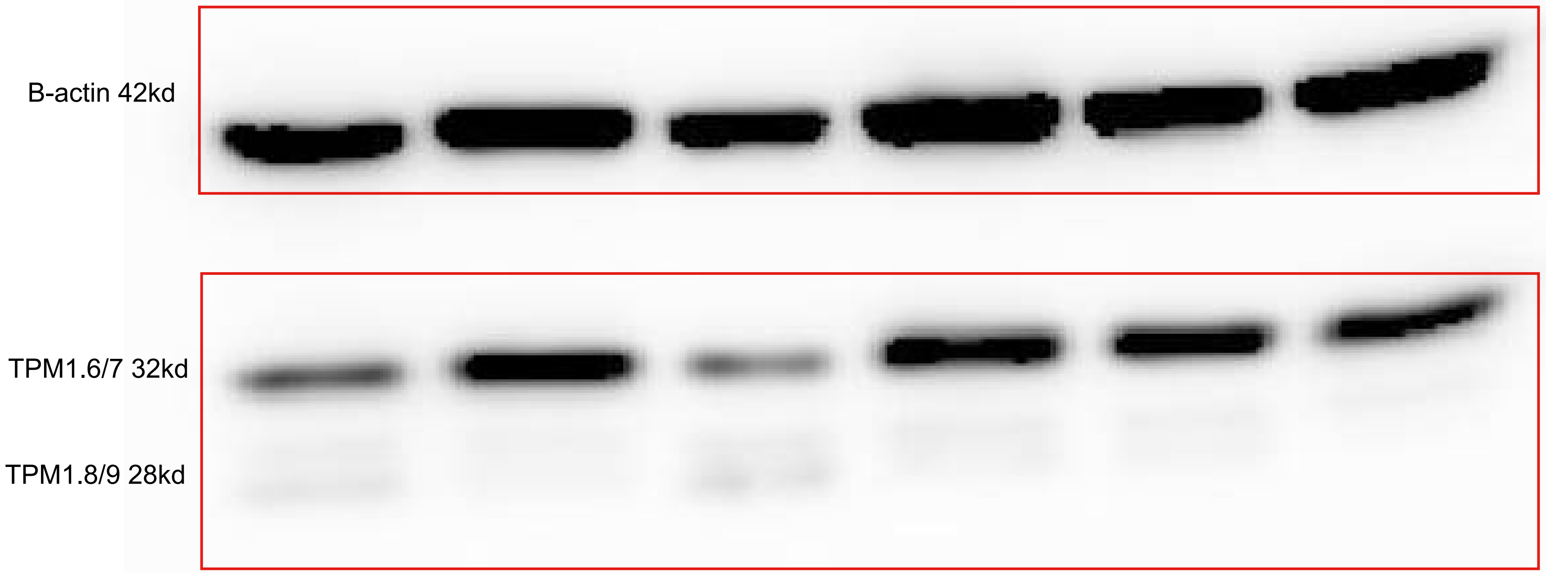

Figure 4B right ESRP1 in COV504

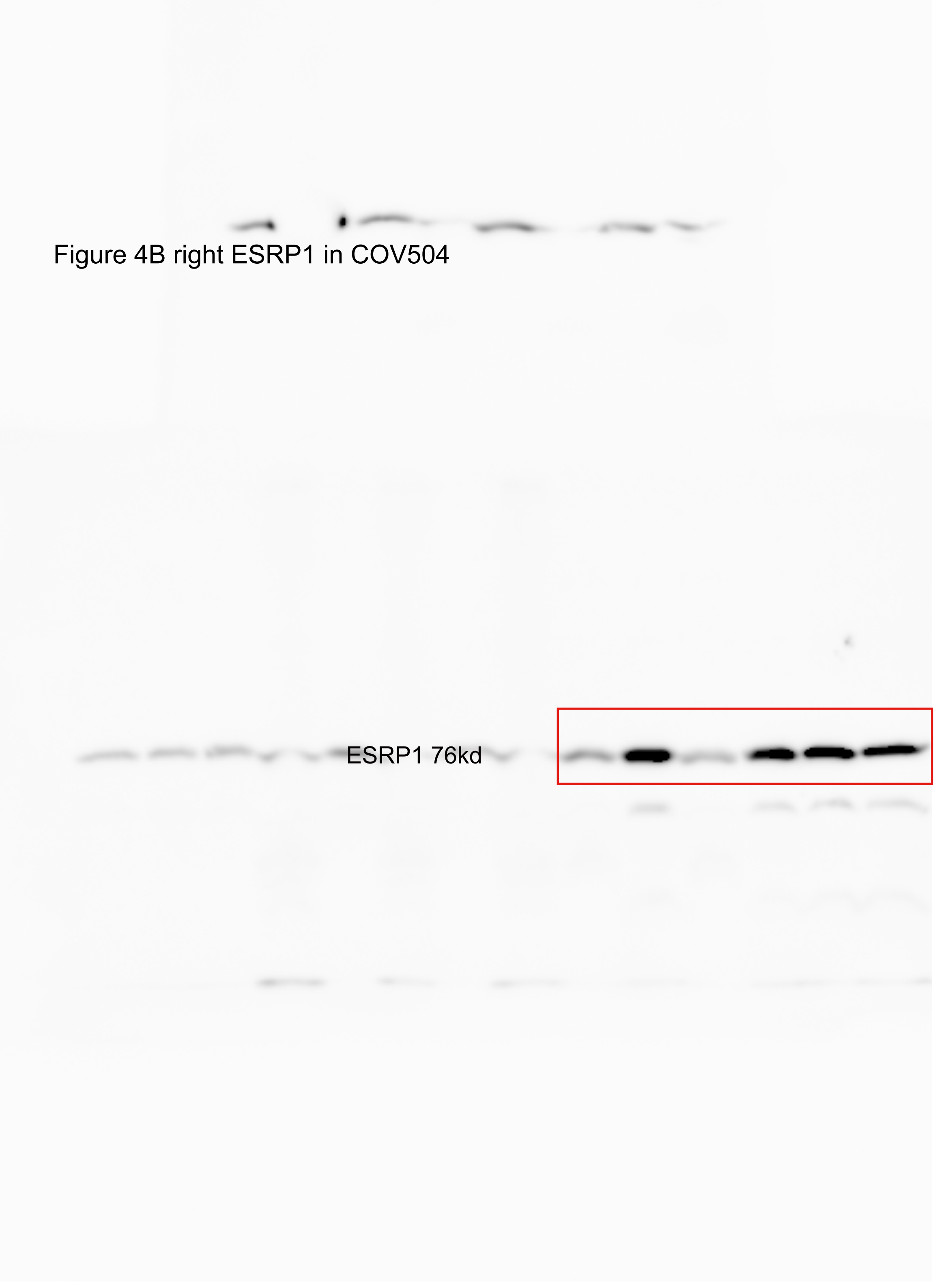

Figure 4B right RBM24 in COV504

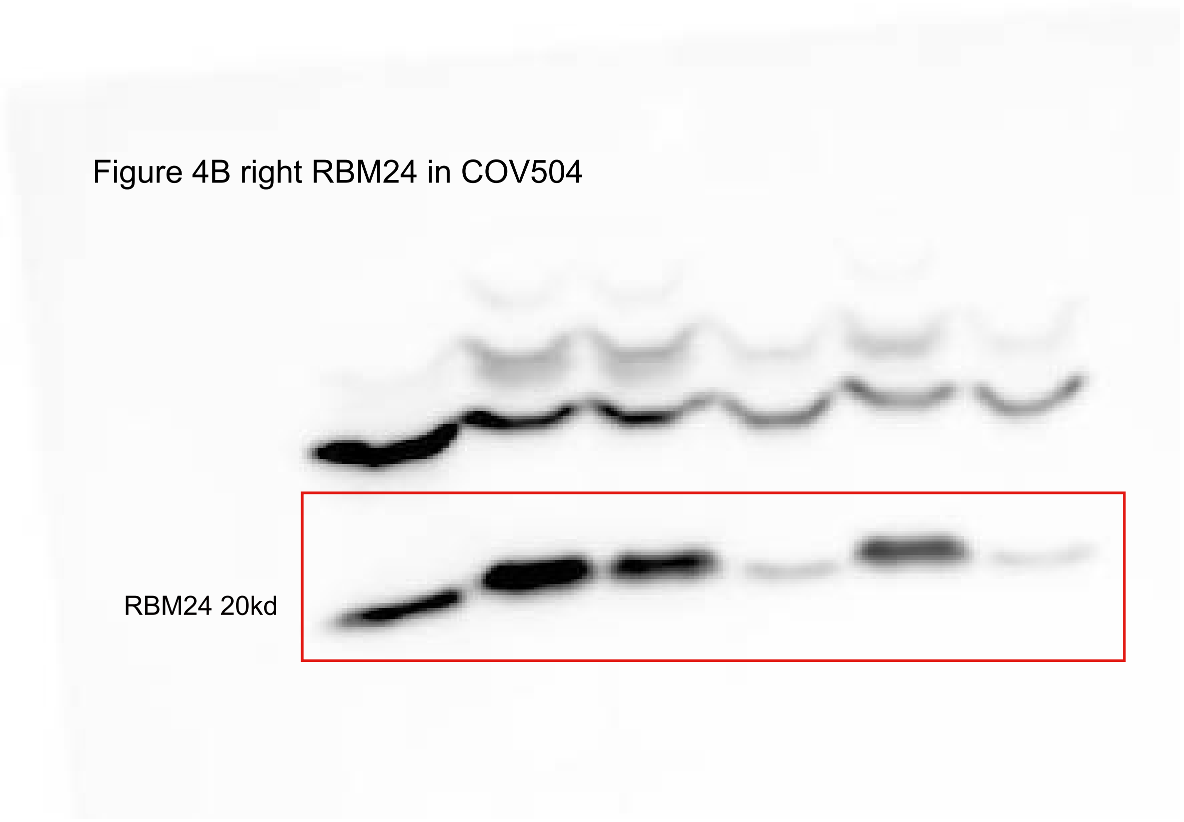

Figure 4B right TPM1 B-actin in COV504

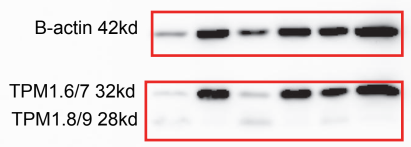

Figure 5B TPM1 B-actin in OV90

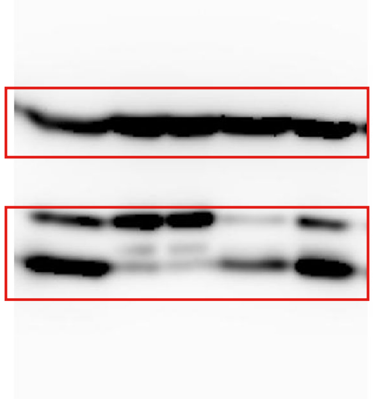

Figure 5B TPM1 B-actin in COV504

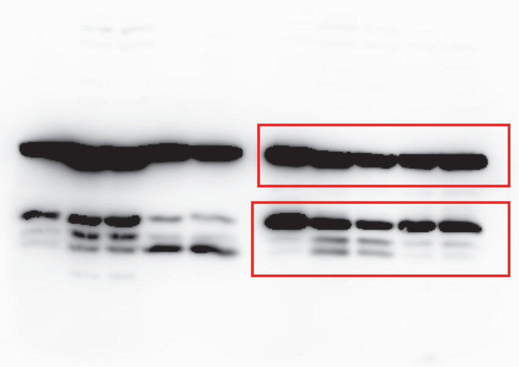

Figure 5B TPM1 B-actin in PEA1

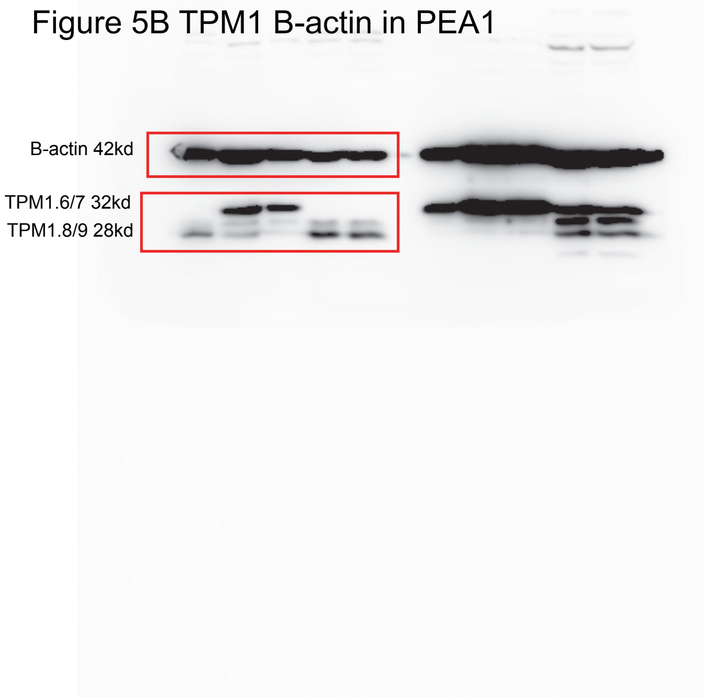

Figure 5B TPM1 B-actin in PEA2

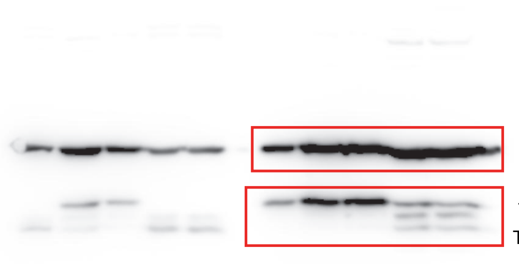

Figure 7 TPM1 B-actin in OV90 and COV504

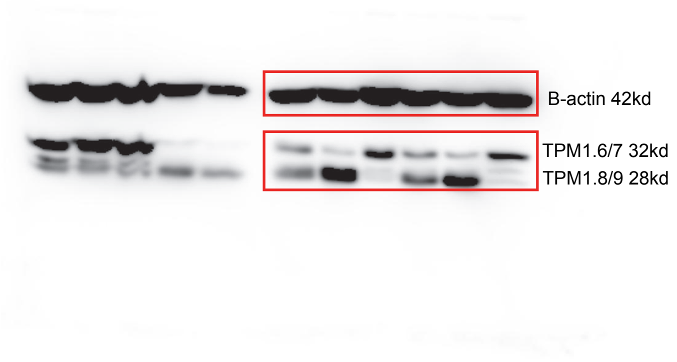

Figure 8A TPM1 B-actin in OV90

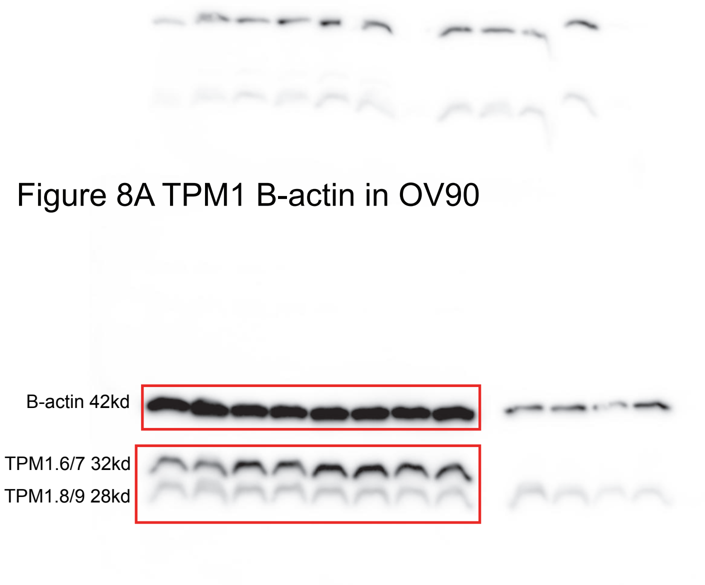

Figure 8B TPM1 B-actin in OV90

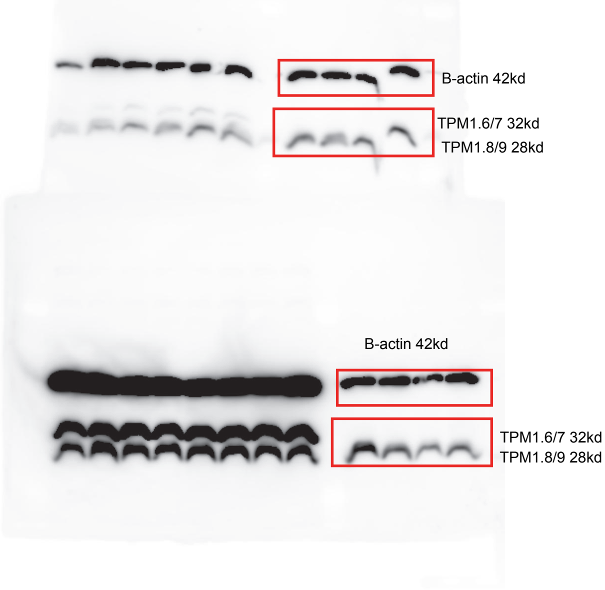

Supplement: Supplementary file 1 — Original data for Blots [file 41418_2024_1267_MOESM1_ESM.pdf]
