# Supplementary material for: Tropomyosin1 isoforms underlie epithelial to mesenchymal plasticity, metastatic dissemination, and resistance to chemotherapy in high-grade serous ovarian cancer
Source: Cell Death Differ. 2024 Feb 16;31(3):360–77. doi: 10.1038/s41418-024-01267-9 (PMC10923901; doi:10.1038/s41418-024-01267-9)
Supplement: Supplementary file 2 — Supplementary Figure Legends [file 41418_2024_1267_MOESM2_ESM.docx]

***Figure legends***

**Fig. 3 - Supplement 1. Isoforms targets in HGSOC.**

**A.** Venn diagrams showing the overlap between ovarian cancer and colon cancer isoforms targets list. The list of isoforms targets is depicted on the right of each Venn diagram. OV90, n=2251; CAOV3, n=2466; HCT116, n=654; SW480, n=541.

**B.** Venn diagrams showing the overlap between ovarian cancer-specific isoforms targets and the EMT hallmark gene list. The list of EMT-related isoforms targets is depicted on the right of each Venn diagram. OV90, n=17; CAOV3, n=30; n=7 overlapped between the two cell lines.

**C.** KEGG pathway analysis of the 40 EMT-related ovarian cancer-specific isoforms targets (and CAOV3-specific AS targets).

**D-F.** Enrichment of Gene Ontology (GO) processes (C. Biological; D. Cellular Components; E. Molecular Functions) relative to the 40 EMT-related ovarian cancer-specific AS targets. Selected GO terms are the top 10 significantly enriched filtered by p value<0.01.

**Fig. 4 - Supplement 1. CD44/EpCAM FACS analysis of RBM24-OE/KD and ESRP1-OE/KD cells.**

**A.** CD44/EpCAM FACS analysis of *RBM24*-OE/KD and *ESRP1*-OE/KD OV90 cells (replica 2). Cells were induced with 1 μg/mL doxycycline for 72 h before analysis. The relative percentages of EpCAM^lo^ and EpCAM^hi^ cells are indicated in each quadrant.

**B.** CD44/EpCAM FACS analysis of *RBM24*-OE/KD and *ESRP1*-OE/KD OV90 cells (replica 3). Cells were induced with 1 μg/mL doxycycline for 72 h before analysis. The relative percentages of EpCAM^lo^ and EpCAM^hi^ cells are indicated in each quadrant.

**C.** Quantitative analysis of EpCAM^lo^ percentage in FACS analysis of *RBM24*-OE/KD and *ESRP1*-OE/KD OV90 cells (replica 1-3). (Means±SD, n=3)

**D.** CD44/EpCAM FACS analysis of *RBM24*-OE/KD and *ESRP1*-OE/KD COV504 cells (replica 2). Cells were induced with 1 μg/mL doxycycline for 72 h before analysis. The relative percentages of EpCAM^lo^ and EpCAM^hi^ cells are indicated in each quadrant.

**E.** CD44/EpCAM FACS analysis of *RBM24*-OE/KD and *ESRP1*-OE/KD COV504 cells (replica 3). Cells were induced with 1 μg/mL doxycycline for 72 h before analysis. The relative percentages of EpCAM^lo^ and EpCAM^hi^ cells are indicated in each quadrant.

**F.** Quantitative analysis of EpCAM^lo^ percentage in FACS analysis of *RBM24*-OE/KD and *ESRP1*-OE/KD COV504 cells (replica 1-3). (Means±SD, n=3)

**Fig. 4 - Supplement 2. Knockdown efficiency of shESRP1 and shRBM24 vectors**

RT-qPCR analysis of ESRP1 and RBM24 expression in sh*RBM24-KD* (knockdown) and sh*ESRP1*-KD (knockdown) OV90 and COV504 ovarian cancer cell line; *GAPDH* expression was employed as control (Means±SD, n=4). There are two shControl vectors and 2-3 sh*RBM24* (knockdown) and sh*ESRP1*-KD (knockdown) used.

**Fig. 4 - Supplement 3.**

**A-B.** Detection of RBP (A) and EMT-TFs (B) binding motifs from the FANTOM database in the TPM1a/b promoter regions.

**C-D.** TPM1a/b reporter vectors were obtained by cloning genomic PCR products encompassing the two promoter sequences as defined in Sevill et al.([53](#_ENREF_53), [54](#_ENREF_54)) in a promoter-less Luciferase plasmid. Transient transfections were conducted on OV90 (A) and COV504 (B) ovarian cancer cell lines (EpCAM^hi^, EpCAM^lo^, and ESRP1- and RBM24-OE cells; Means±SD, n=4). P values are relative to the comparison with the parental cell lines.

**E-H.** TPM1a/b reporter vectors were obtained by cloning genomic PCR products encompassing the two promoter sequences as defined in Sevill et al.([53](#_ENREF_53), [54](#_ENREF_54)) in a promoter-less Luciferase plasmid. Transient transfections were conducted on OV90 (C-D) and COV504 (E-F) ovarian cancer cell lines (EpCAM^hi^, EpCAM^lo^, and ESRP1- and RBM24-KD cells; Means±SD, n=4). P values are determined by comparison with the parental, EpCAM^hi^ orEpCAM^lo^ cell line.

**Fig. 6 - Supplement 1. TOP-Flash luciferase reporter analysis of Wnt signaling activity.**

TOP-Flash luciferase reporter analysis of Wnt signaling activity in *Tpm1.6/7/8/9*-OE (upper histogram) and upon knockdown by siRNA of *Tpm1.6/7* and *Tpm1.8/9* in COV504 and PEA2 cells. P values are relative to the comparison with the parental cell lines (Means±SD, n=4-5).

**Fig. 7 - Supplement 1.**

Kaplan-Meier analysis of overall survival based on the relative expression of the *TPM1* gene, and its Tpm1.6/7/8/9 isoforms.

**Fig. 7 - Supplement 2. EpCAM^lo^ , ZEB1 and *Tpm1.8/9* isoforms confer resistance to platinum- and taxane-based therapies.**

**A.** Dose-response curves relative to EpCAM^hi^ and EpCAM^lo^ subpopulations of OV90 and COV504 grown in the presence of different concentrations of paclitaxel and cisplatin (log scale and cell viability on the x and y axis, respectively). IC_50_ values were calculated from biological quadruplicates for each experiment (Means±SD, n=4).

**B.** Dose-response curves relative to *ZEB1*-shRNA induced OV90 and COV504 grown in the presence of different concentrations of paclitaxel and cisplatin (log scale and cell viability on the x and y axis, respectively). IC_50_ values were calculated from biological quadruplicates for each experiment (Means±SD, n=4).

**C.** Dose-response curves relative to *Tpm1.6/7/8/9*-OE CAOV3 cells grown in the presence of different concentrations of paclitaxel and cisplatin (log scale and cell viability on the x and y axis, respectively). IC_50_ values were calculated from biological quadruplicates for each experiment (Means±SD, n=4).

**D.** Dose-response curves of si*Tpm1.6/7* and si*Tpm1.8/9* knockdown CAOV3 cells cultured in the presence of different concentrations of paclitaxel (left) and cisplatin (right) concentrations (log scale and cell viability on the x and y axis, respectively). IC_50_ values were calculated from biological quadruplicates for each experiment (Means±SD, n=4).

**Fig. 8 - Supplement 1.**

**A.** Compound # 1 and #3 cause the removal of Tpm1.8/1.9 from the lamellipodium of human fibroblasts. Human immortalized BJeH fibroblasts were plated onto glass coverslips for 24 h and then exposed to DMS0 (up) or 10 μM compound #1 or #3 (middle and down) dissolved in DMSO for a further 24 h. The cells were then fixed and Tpm1.8/1.9 visualized using a rat monoclonal antibody exactly as described in Brayford et al. (2016). Arrowheads identify lamellipodia in (up) control cells positive for Tpm1.8/9 and (middle and down) lamellipodia negative for Tpm1.8/9 in cells exposed to compound #1 or 3..

**B.** Compound #1 and #3 causes the removal of Tpm1.8/1.9 from the lamellipodium of OV90 cells. OV90 cells were plated onto glass coverslips for 24 h and then exposed to DMS0 (up) or 10 μM (middle) compound #1 and 3 (down) dissolved in DMSO for a further 24 h. Arrowheads identify lamellipodia in (up) control cells positive for Tpm1.8/9 and (middle and down) lamellipodia negative for Tpm1.8/9 in cells exposed to compound # 1 or #3.

**Fig. 8 - Supplement 2.**

**A.** RT-qPCR analysis of *TPM1* isoforms and EMT-related gene expression in OV90 parental cells cultured for 24 hrs. in the presence of compound #1 and #3 at 0, 2, 5, 10 and 20 μM. The values were calculated by normalizing with the untreated cells. P values < 0.05 are shown by red bars while grey bars indicate lower values; GAPDH expression was employed as control (Means±SD, n=4).

**B.** RT-qPCR analysis of *TPM1* isoforms and EMT-related gene expression in OV90 parental cells cultured for 48 hrs. in the presence of compound #1 or #3 at 0, 2, 5, 10 and 20 μM. The values were calculated by normalizing with the untreated cells. P values < 0.05 are shown by red bars while grey bars indicate lower values; GAPDH expression was employed as control (Means±SD, n=4).

**C.** Dose-response curves of parental OV90 cells treated with compound #1 or #3 in the presence of different cisplatin concentrations. IC_50_ values were calculated from biological triplicates for each experiment (Means±SD, n=4).

**D.** Dose-response curves of parental OV90 cells treated with compound #1 or #3 in the presence of different paclitaxel concentrations. IC_50_ values were calculated from biological triplicates for each experiment (Means±SD, n=4).

**E.** RT-qPCR analysis of *TPM1* isoforms and EMT-related gene expression in OV90 EpCAM^lo^ cells cultured for 24 hrs. in the presence of compound #1 or #3 at 0, 2, 5, 10 and 20 μM. The values were calculated by normalizing with the untreated cells. P values < 0.05 are shown by red bars while grey bars indicate lower values; GAPDH expression was employed as control (Means±SD, n=4).

**F.** RT-qPCR analysis of *TPM1* isoforms and EMT-related gene expression in OV90 EpCAM^lo^ cells cultured for 48 hrs. in the presence of compound #1 or #3 at 0, 2, 5, 10 and 20 μM. The values were calculated by normalizing with the untreated cells. P values < 0.05 are shown by red bars while grey bars indicate lower values; GAPDH expression was employed as control (Means±SD, n=4).

**G.** Dose-response curves of OV90 EpCAM^lo^ cells treated with compound #1 or #3 in the presence of different cisplatin concentrations. IC_50_ values were calculated from biological triplicates for each experiment (Means±SD, n=4).

**H.** Dose-response curves of OV90 EpCAM^lo^ cells treated with compound #1 or #3 in the presence of different paclitaxel concentrations. IC_50_ values were calculated from biological triplicates for each experiment (Means±SD, n=4).
