# Supplementary figures and images for: Tropomyosin1 isoforms underlie epithelial to mesenchymal plasticity, metastatic dissemination, and resistance to chemotherapy in high-grade serous ovarian cancer
Source: Cell Death Differ. 2024 Feb 16;31(3):360–77. doi: 10.1038/s41418-024-01267-9 (PMC10923901; doi:10.1038/s41418-024-01267-9)

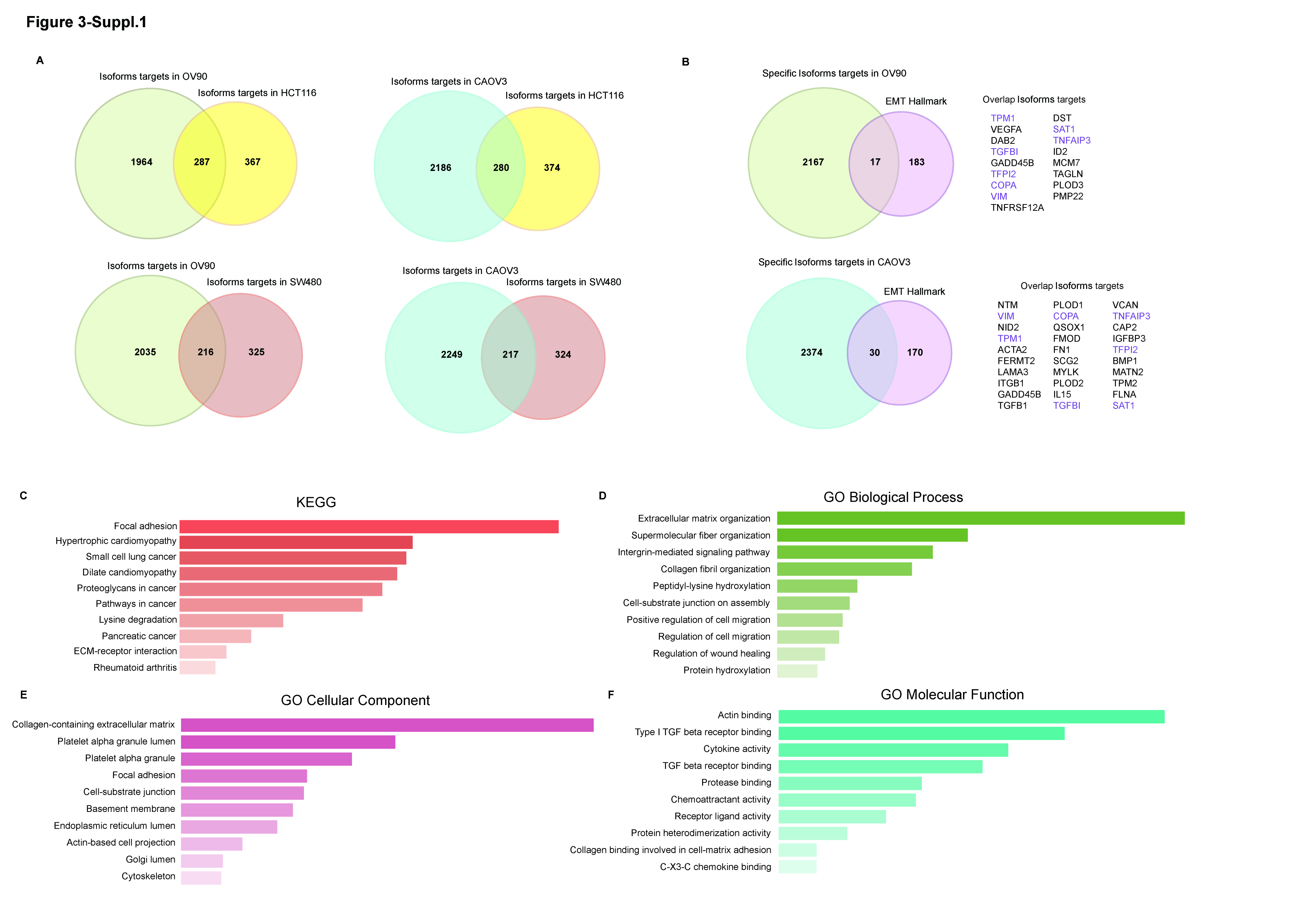

Supplement: Supplementary file 3 — Fig.3-Suppl1 [file 41418_2024_1267_MOESM3_ESM.tif]

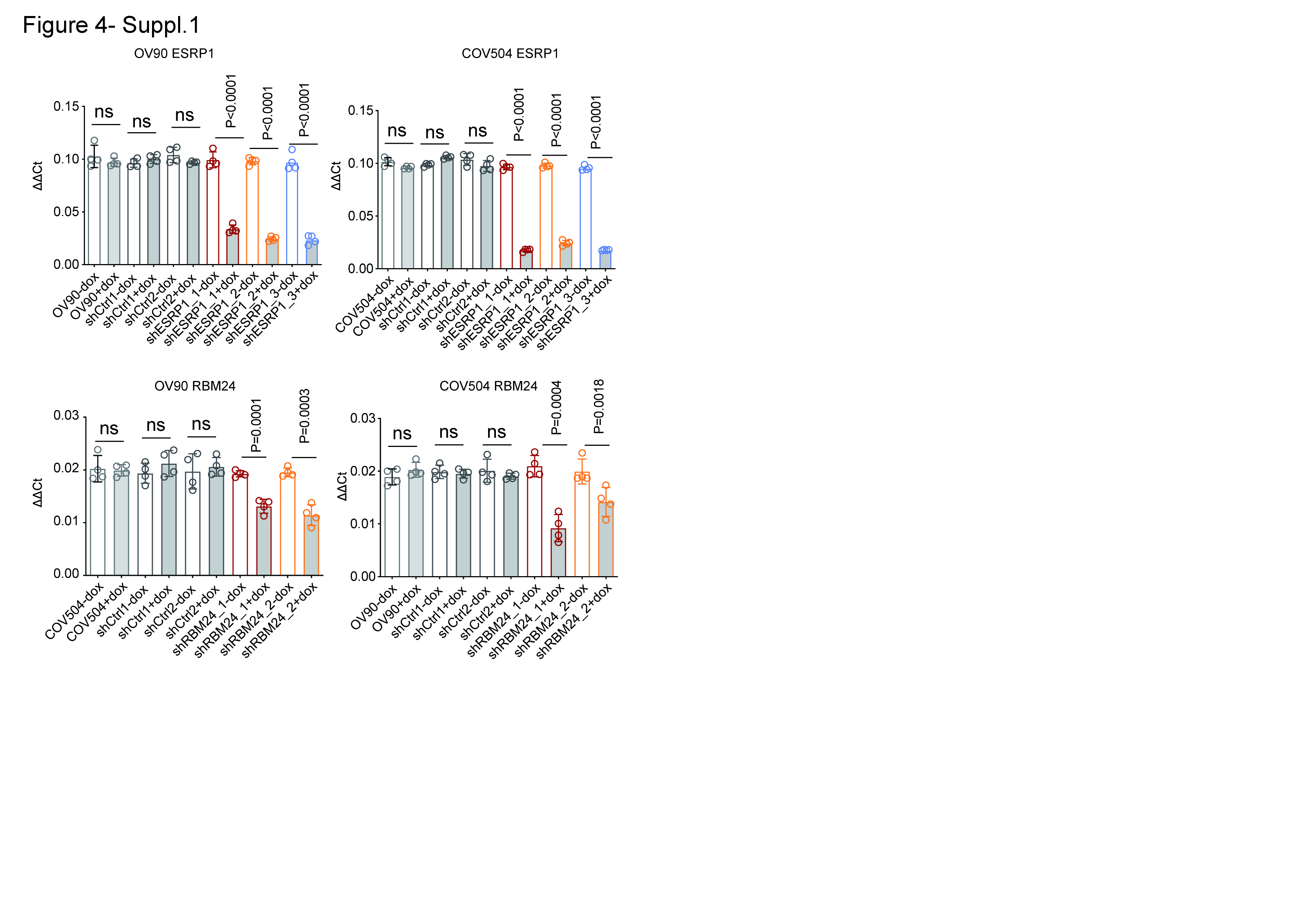

Supplement: Supplementary file 4 — Fig.4-Suppl1 [file 41418_2024_1267_MOESM4_ESM.tif]

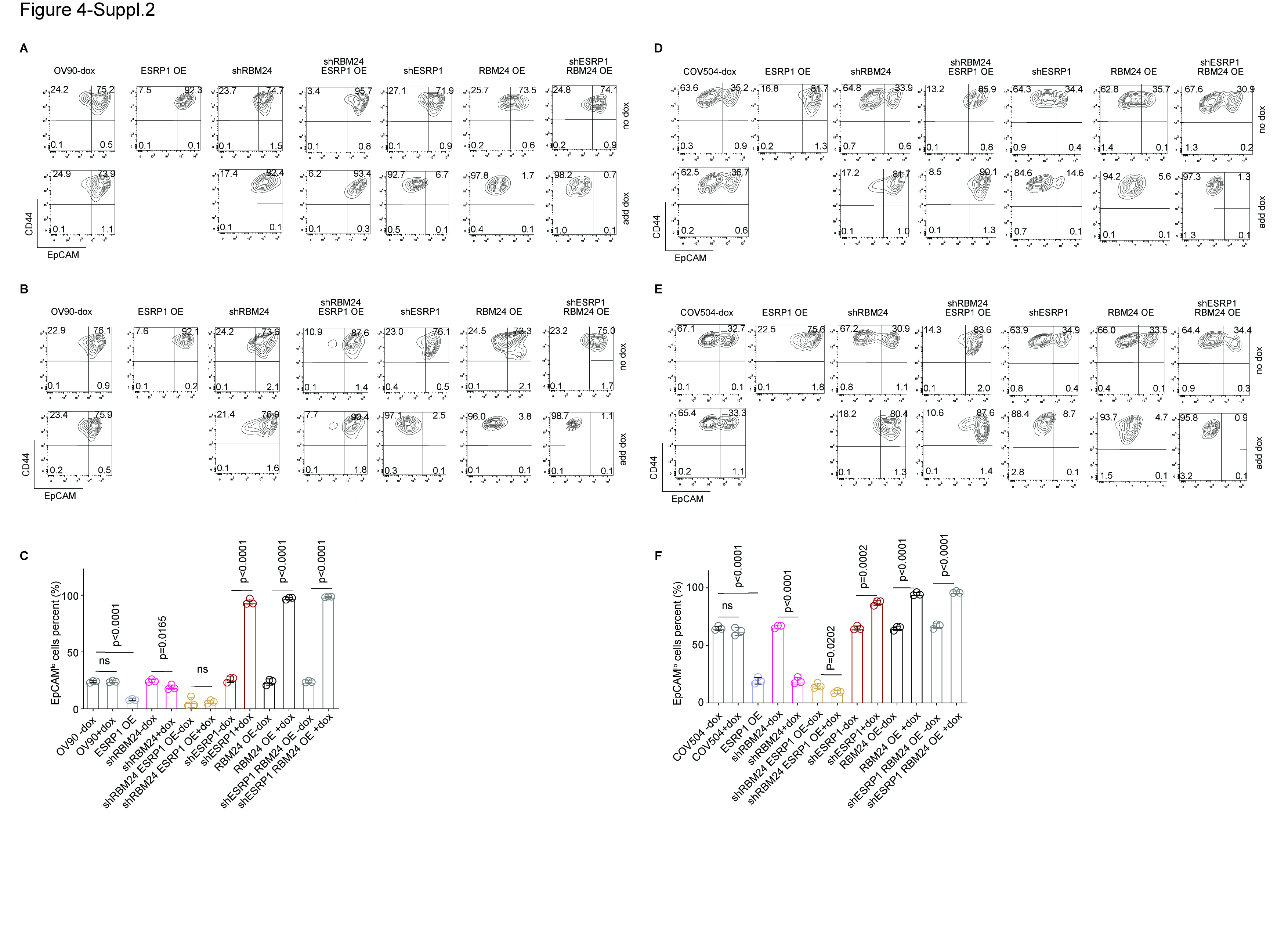

Supplement: Supplementary file 5 — Fig.4-Suppl2 [file 41418_2024_1267_MOESM5_ESM.tif]

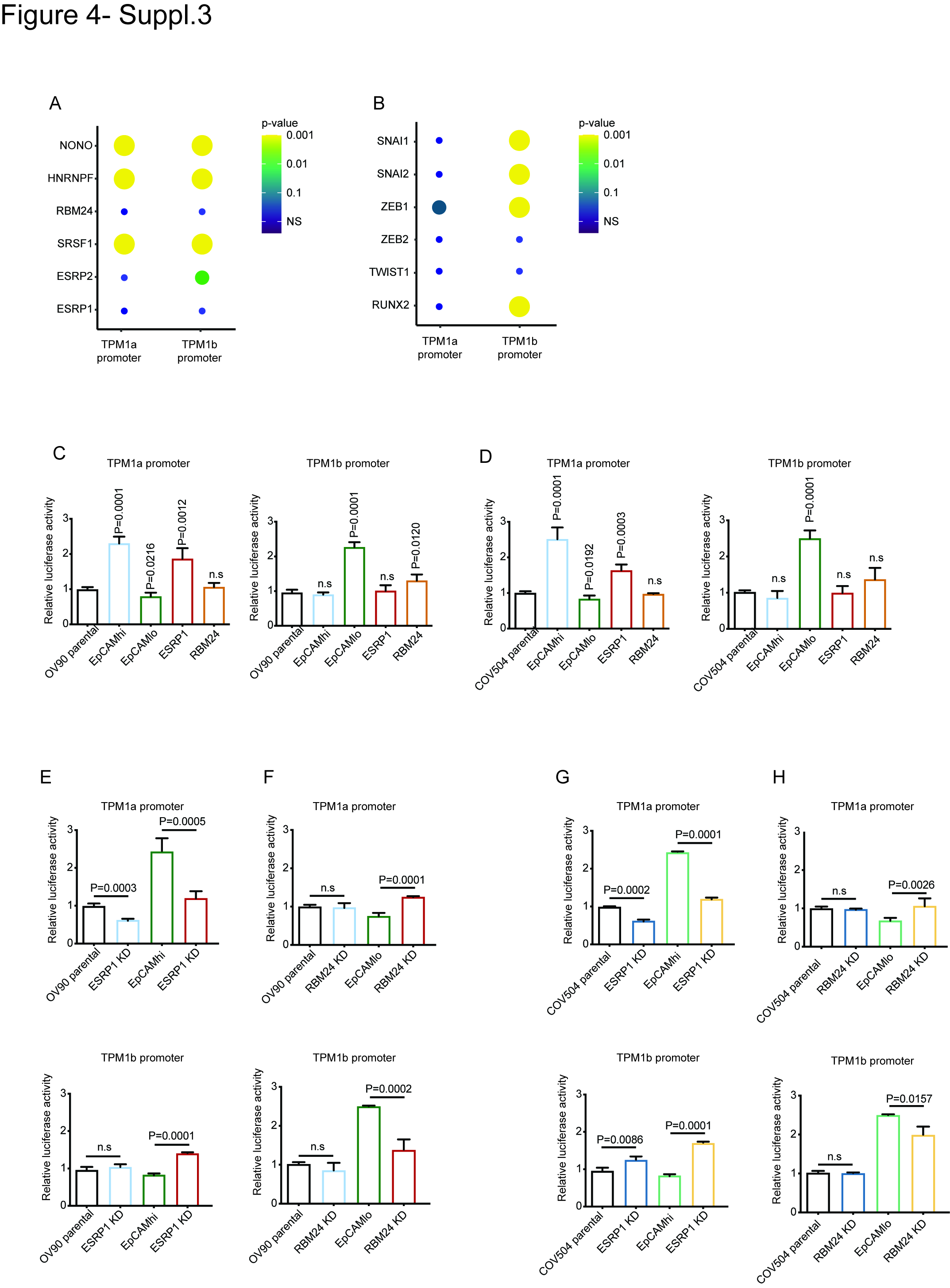

Supplement: Supplementary file 6 — Fig.4-Suppl3 [file 41418_2024_1267_MOESM6_ESM.tif]

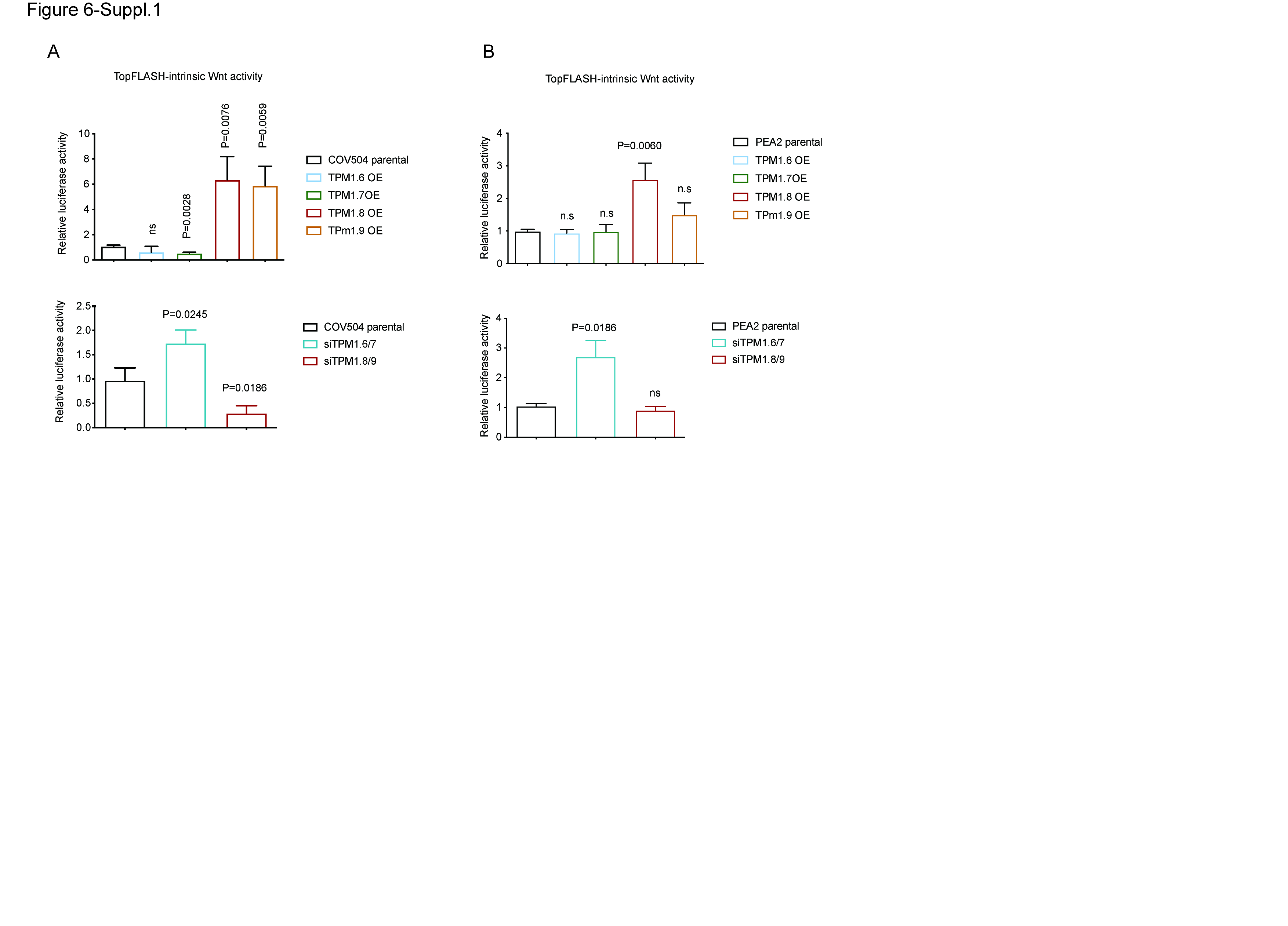

Supplement: Supplementary file 7 — Fig.6-Suppl1 [file 41418_2024_1267_MOESM7_ESM.tif]

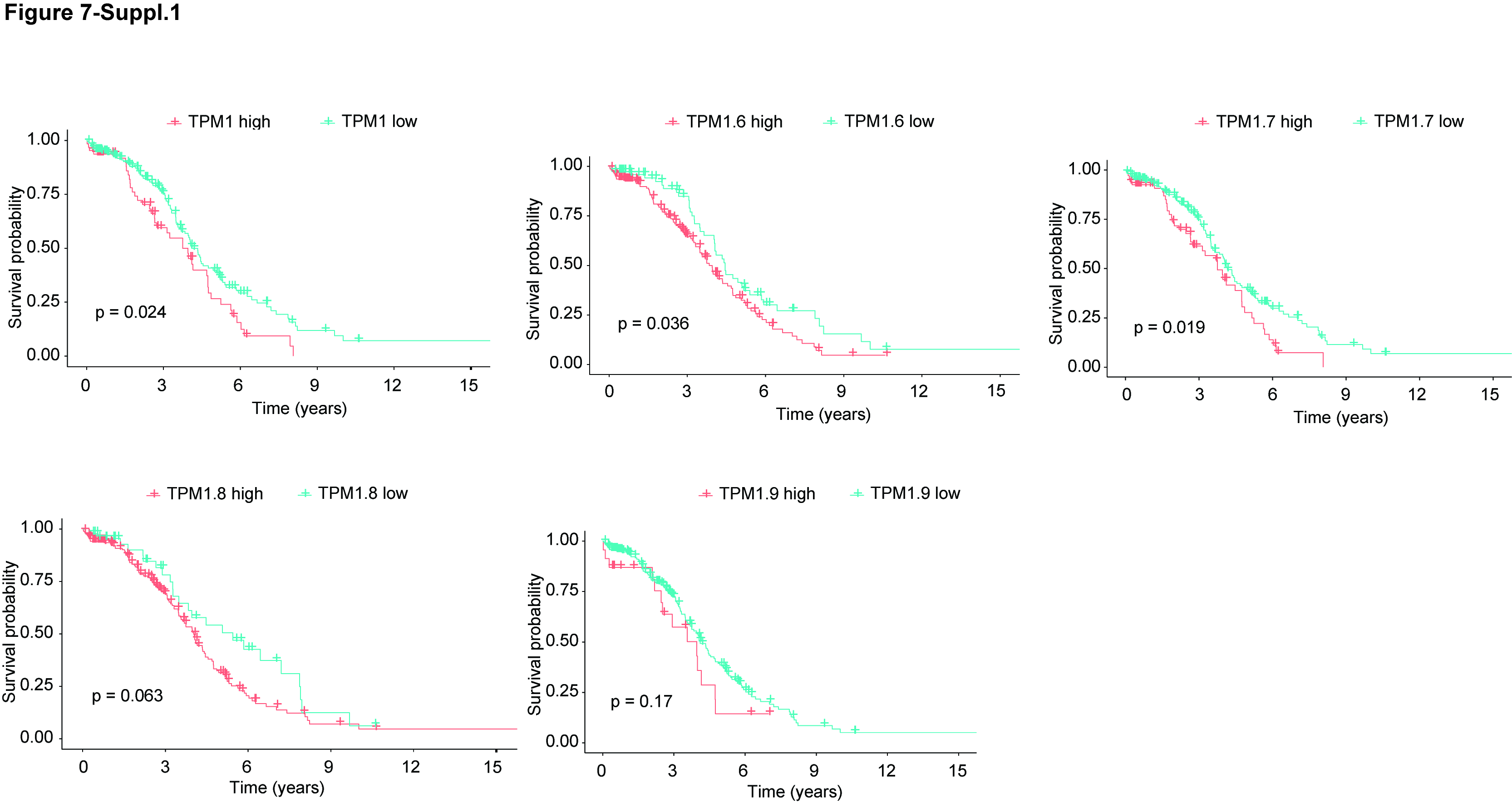

Supplement: Supplementary file 8 — Fig.7-Suppl1 [file 41418_2024_1267_MOESM8_ESM.tif]

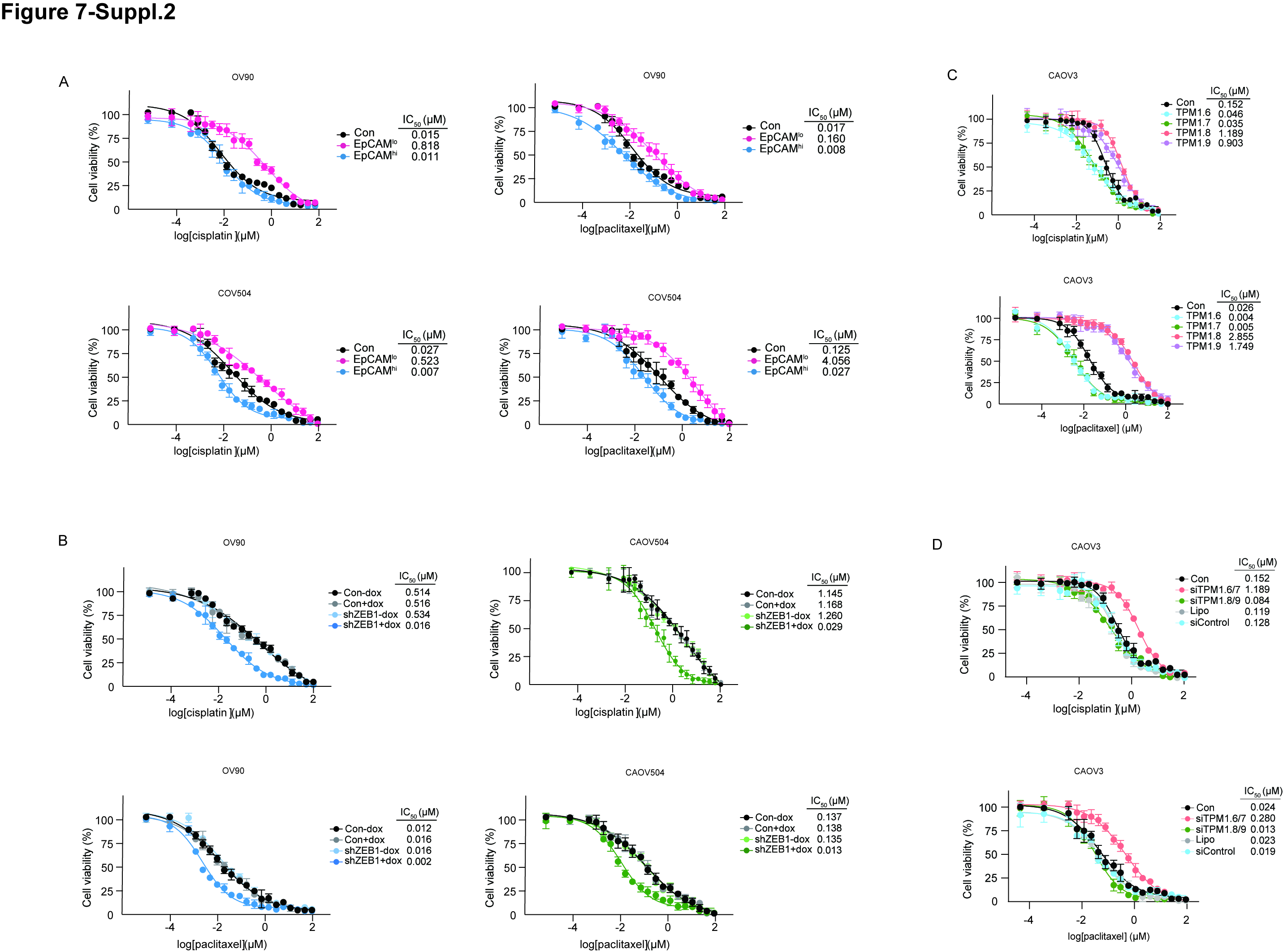

Supplement: Supplementary file 9 — Fig.7-Suppl2 [file 41418_2024_1267_MOESM9_ESM.tif]

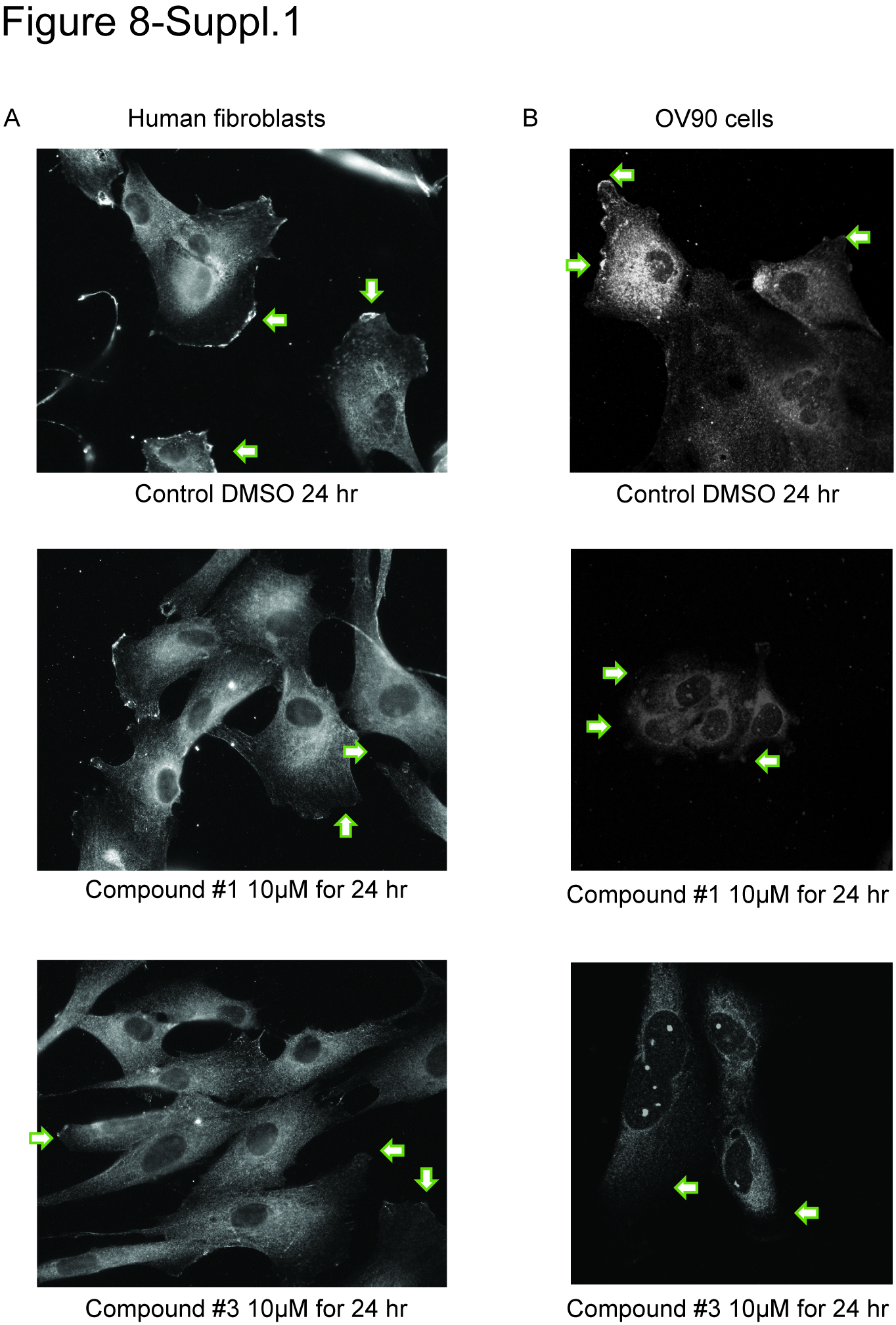

Supplement: Supplementary file 10 — Fig.8-Suppl1 [file 41418_2024_1267_MOESM10_ESM.tif]

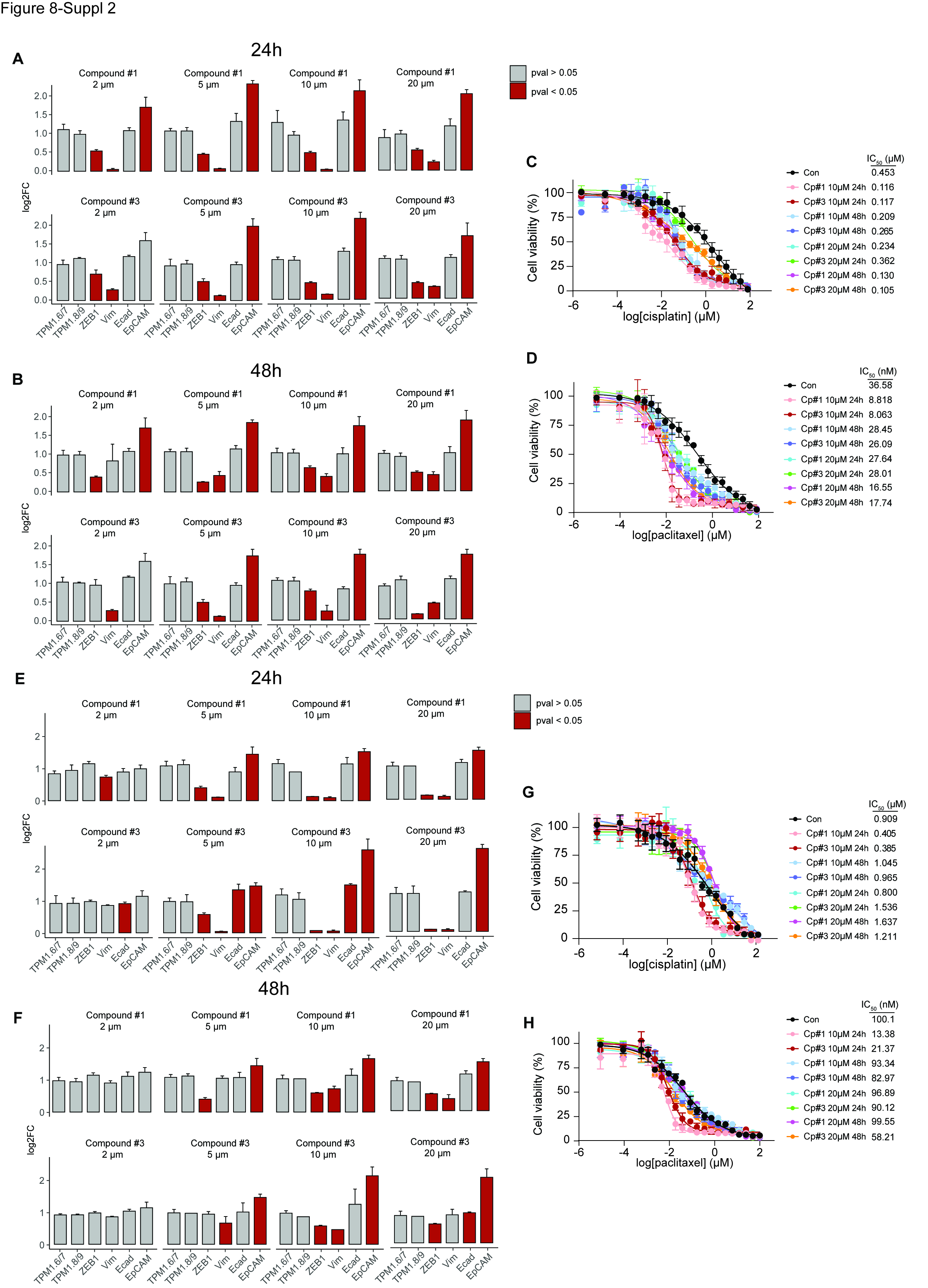

Supplement: Supplementary file 11 — Fig.8-Suppl2 [file 41418_2024_1267_MOESM11_ESM.tif]
